# The complexity of simple counting: ERP findings reveal early perceptual and late numerical processes in different arrangements

**DOI:** 10.1038/s41598-022-10206-y

**Published:** 2022-04-26

**Authors:** Shadi Akbari, Mojtaba Soltanlou, Hassan Sabourimoghaddam, Hans-Christoph Nuerk, Hartmut Leuthold

**Affiliations:** 1 Cognitive Neuroscience Lab, Department of Psychology, University of Tabriz, Tabriz, Iran; 2 Department of Psychology, University of Tuebingen, Schleichstreet 4, 72076 Tuebingen, Germany; 3 School of Psychology, University of Surrey, Guildford, UK; 4 Leibniz-Institut Für Wissensmedien, Tuebingen, Germany; 5 LEAD Graduate School and Research Network, University of Tuebingen, Tuebingen, Germany

**Keywords:** Perception, Electroencephalography - EEG

## Abstract

The counting process can only be fully understood when taking into account the visual characteristics of the sets counted. Comparing behavioral data as well as event-related brain potentials (ERPs) evoked by different task-irrelevant arrangements of dots during an exact enumeration task, we aimed to investigate the effect of illusory contour detection on the counting process while other grouping cues like proximity were controlled and dot sparsity did not provide a cue to the numerosity of sets. Adult participants (N = 37) enumerated dots (8–12) in irregular and two different types of regular arrangements which differed in the shape of their illusory dot lattices. Enumeration speed was affected by both arrangement and magnitude. The type of arrangement influenced an early ERP negativity peaking at about 270 ms after stimulus onset, whereas numerosity only affected later ERP components (> 300 ms). We also observed that without perceptual cues, magnitude was constructed at a later stage of cognitive processing. We suggest that chunking is a prerequisite for more fluent counting which influences automatic processing (< 300 ms) during enumeration. We conclude that the procedure of exact enumeration depends on the interaction of several perceptual and numerical processes that are influenced by magnitude and arrangement.

## Introduction

The development of the ability to count has a crucial effect on daily life and academic skills^1^ and is a predictive factor of a child’s progress in mathematical skill acquisition^2^. Since counting is mostly performed in response to visual stimuli, visual perception has a crucial role in this process^3^. Therefore, recently more attention has been devoted to investigating the effects of visual stimulus properties on non-symbolic number processing and exact enumeration^4^. Previous research has revealed that enumeration is influenced by aspects of visual stimuli such as contour length^5^ and cumulative surface area^6^, as well as clustering and stimulus diameter^7,8^. Thus, there is now strong evidence for the view that visual aspects affect non-symbolic number processing and that numbers are not perceived independently from the visual characteristics of the stimuli^9^. One of the most important characteristics of a visual counting stimulus is its spatial arrangement.

From previous behavioral studies we know about the influence of different spatial arrangements on the counting process^10–14^. The results of these studies showed a higher rate of exact enumeration with regular arrangements. What makes counting faster in regular arrangements? Some authors believe that the time benefit of counting regular arrangements is due to *grouping*^13,14^, especially if the sets are grouped into small subsets in the subitizing range^15^. Subitizing is defined as the fast and accurate enumeration of up to about 3 or 4 objects^16^. It has been argued that grouping and subitizing are the main components of the enumeration process^13,17,18^. According to Bourdon (1908), number perception consists of at least two mental processes: first, the perception of the units of which the total number is composed and, second, the grouping of these units.

**Figure 1:**
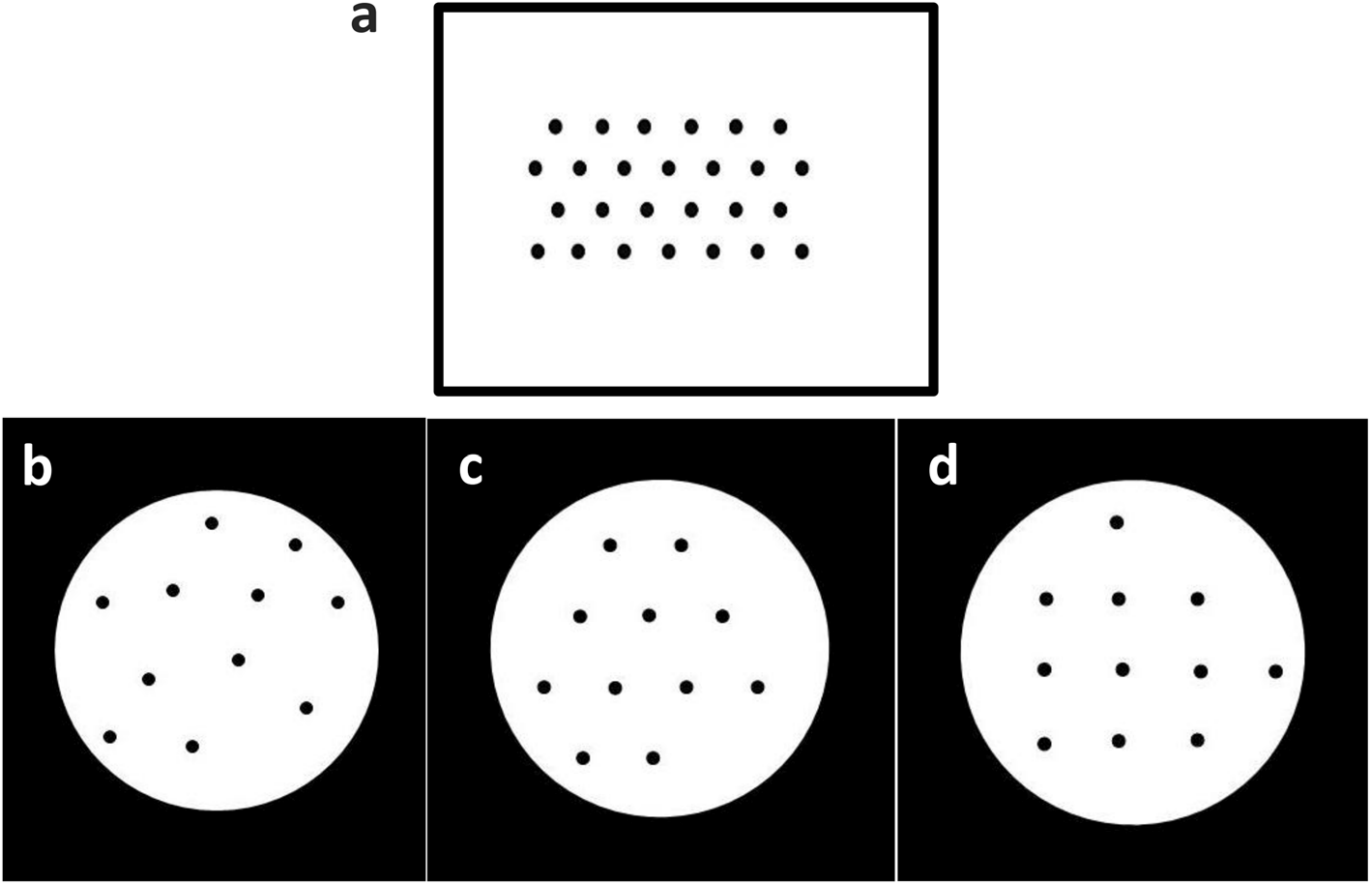
(**a**) Sample of an arrangement without proximity and similarity effect. (**b**) irregular arrangement, (**c**) hexagonal arrangement, and (**d**) quadrangular arrangement for number 11.

As important grouping factors, proximity and similarity laws of Gestalt have been extensively studied^18–20^. Proximity has been viewed as the main cause of grouping and subitizing^10,18,21^. According to this law, objects or events that are near to one another (in space or time) are perceived as forming a group. Similarity, especially in the sense of shape and color, is another factor which facilitates grouping^22^. The effect of grouping on numerosity estimation has been investigated in some previous behavioral and electrophysiological studies. As an example, He and colleagues (2015) reported that connectivity of dots is effective in numerosity judgments, while grouping by similarity (color) is not^23,24^. According to electrophysiological findings, proximity affects early visual processing as indicated by modulations of the visual P1 component, whereas similarity has a later effect on the ERP waveform and hence information processing^25^. However, not all arrangements show the effects of proximity and similarity, for instance, when individual items are spaced by an equal distance from each other and have the same shape and color (see Fig. 1a).

It is assumed that dense dot patterns give rise to Kanizsa subjective edges, which are mental lattices created by the lines that subjects imagine between dots, which are also known as illusory contours^26^. This assumption was corroborated by the finding that the probability of perceiving these illusory contours decreases with increasing distance between dots, although it never drops to zero^27^. Moreover, based on their findings, Kubovy, Holcombe, and Wagemans (1998) proposed that there are various grouping strategies depending on the type of arrangement. These authors investigated the grouping phenomenon by presenting illusory dot lattices, although not in a counting task. They showed 16 different regular arrangements which differed in their illusory lattices. The most stable lattice in terms of the orientation in which the lattice was grouped was an arrangement with elongated rectangles. In contrast, an arrangement with a hexagonal lattice, which is characterized by equal distances between dots, was found to be the most ambiguous with regard to grouping. According to the authors, this ambiguity implies that subjects use various grouping strategies. Therefore, illusory lattice formation is another cause of grouping. Considering its definition, illusory lattice formation is different from similar Gestalt grouping principles such as “good continuation” which is a tendency to perceive a line as continuing its established direction^28^.

We now have a better understanding of the perceptual processing of illusory contours. According to neuroimaging and neurophysiological studies, two basic mechanisms might be critically involved in illusory contour perception. One is the fast-local low-level mechanism, related to early visual processing areas (V1/V2) and an early time interval (about 100 ms post-stimulus). The other is the late-global high-level mechanism which involves higher visual areas and later time intervals of visual perception (about 200–300 ms post-stimulus)^29^. It is mentioned that the illusory contour detections caused by proximity effect, affects early visual or fast-local processing in V1/V2, at 100–120 ms post-stimulus^30^. This process is followed by forwarding the segmented features from the illusory figures to higher cortical areas in temporal and parietal regions for figure recognition and integrational processing^31^. This later stage is attributed to late-global high- level mechanism which takes place around 200 ms post-stimulus^29^. Grouping is strongly influenced by the presence of figures defined by illusory contours^32^. However, still it remains unclear which of the above mechanisms (if any) has a determining role in the grouping process during a counting task, especially when chunking strategies are changed by arrangement modulations.

To our knowledge, the effect of illusory contours on enumeration has not yet been investigated. Moreover, it should be remembered that figure organization and perceptual grouping depends on both task^33^ and individual processing strategies^34^.

How illusory contours affect the counting process is an important unresolved research question. Since grouping by illusory contours is mostly modulated by arrangement changes, we must study counting in different arrangements in such a way that the effect of other influential factors of grouping like proximity and similarity are controlled in order to investigate their pure effect. Therefore, in this exploratory study we addressed the following: (i) whether or not the regularity of arrangements (manipulated by their illusory contours) influences the counting process and (ii) if so, which processing stage(s) are modulated by different spatial arrangements in the absence of proximity and similarity cues during exact enumeration. An answer to these questions is important not only for research focusing on the basic cognitive mechanisms underlying enumeration but also for research concerned with visual perception and grouping. We examined the effect of arrangement on behavioral data and ERPs during an exact enumeration task in an attempt to address these questions. Given the exploratory nature of our study, we searched for the effect of arrangement on information processing within the brain in terms of both location and time. Three types of arrangement were employed, two regular ones differing in lattice formation and an irregular one. In addition, since participants performed an exact enumeration task, number magnitude was manipulated, that is, the different arrangements consisted of 8 to 12 dots. This latter manipulation was assumed to produce a size effect on behavioral performance, that is, enumeration rate decreased with increasing magnitude. We had no specific predictions about how magnitude would affect ERPs. Furthermore, since according to previous neuroimaging studies the two hemispheres are activated differently by counting and contour detection^35–37^, whenever we found a bilateral effect, hemisphere was considered in the data analysis to see if and how enumeration and contour detection differentially influenced lateralized activity in the brain. Crucially, with regard to our first research question, we assumed that the type of spatial arrangement influences exact enumeration processing in such a way that the enumeration rate is higher for regular than for irregular arrangements. Concerning our second research question, we investigated whether this was an early or a late effect or, as defined by Seghier et al., (2006), a fast-local or a late-global effect. We hypothesized that if the arrangement type influences early perceptual processing during an exact enumeration task, this should be reflected by the ERP waveform before 300 ms, whereas its effect on higher-level, conceptual number processing should be reflected later in time.

## Results

### Behavioral and subjective responses

**Figure 2:**
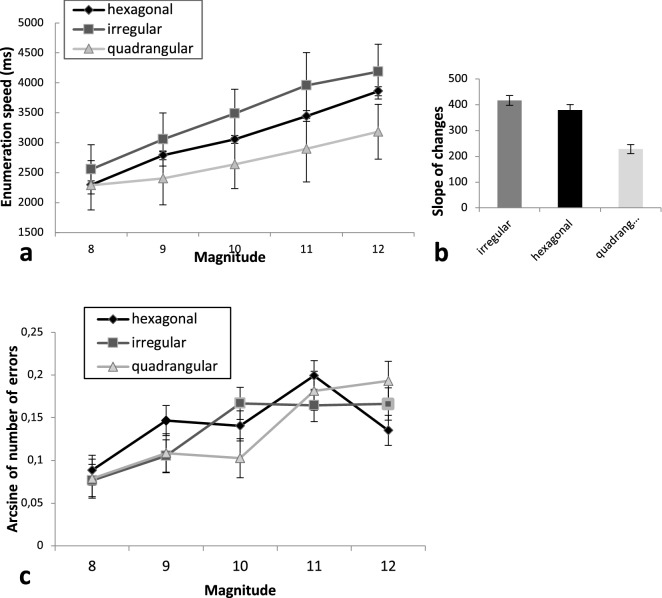
(**a**) Mean reaction times in three different arrangements. (**b**) Slope of reaction time changes by increased magnitude in three arrangements. (**c**) Interaction between magnitude and arrangement in error rates. All error bars depict 1 SE of M.

Information on descriptive statistics for all of the variables is available in Table S1 in the Supplementary Material. A repeated measure analysis of variance (ANOVA) of reaction times (RTs) showed a significant main effect of arrangement, [Greenhouse–Geisser adjusted *F*(2, 74) = 84.74, *p* < 0.001, ε = 0.772*, ɳ*_*p*_^*2*^ = 0.70], Pairwise comparison revealed significant difference between all arrangements [*p* < 0.001], while the shortest RT was for the quadrangular arrangement, and the longest RT was for the irregular arrangement. The significant main effect of magnitude, [Greenhouse–Geisser adjusted *F*(4, 148) = 234.03, *p* < 0.001, ε = 0.682, *ɳ*_*p*_^*2*^ = 0.86], reflected the RT increase with magnitude. In pairwise comparison, the largest difference was found between numbers 8 and 12, [MD = -1363.76, *p* < 0.001]. The Arrangement × Magnitude interaction was also significant, [*F*(8, 296) = 18.31, *p* < 0.001, *ɳ*_*p*_^*2*^ = 0.32] (see Fig. 2a).

In order to uncover the Arrangement × Magnitude interaction, the slopes of RT changes across magnitudes were separately calculated for the different arrangements. To this end, RT changes in response to the increased magnitude in each arrangement were considered and the slope (unstandardized beta coefficient) was calculated by a standard linear regression, where RT was regressed on the numbers of dots (8–12) for each arrangement separately. The main effect of arrangement on this slope was significant, [*F*(2, 70) = 53.92, *p* < 0.001, *ɳ*_*p*_^*2*^ = 0.60], showing a smaller RT increase with magnitude in the case of the quadrangular arrangement than for the two other arrangements. Pairwise comparison revealed a significantly smaller increase in RT from small to large numbers for the quadrangular arrangement compared to irregular [*MD* = − 188.66, *p* < 0.001] and hexagonal [*MD* = − 150.72, *p* < 0.001] arrangements (cf. Fig. 2b). The slope was not significantly different between the hexagonal and irregular arrangements [*MD* = − 37.93, *p* = 0.226].

To investigate the effect of our main factors (Arrangement and Magnitude) on counting accuracy, a repeated measure ANOVA was performed on the arcsine-transformed error rates. A significant main effect of magnitude was found [*F*(4, 140) = 10.30, *p* < 0.001, *ɳ*_*p*_^*2*^ = 0.23], showing that exact enumeration was more error prone with increasing magnitude. No significant main effect of arrangement was observed, [*F*(2, 70) = 0.44, *p* = 0.697, *ɳ*_*p*_^*2*^ = 0.01], however the Arrangement × Magnitude interaction was significant, [*F*(8, 280) = 2.19, *p* = 0.028, *ɳ*_*p*_^*2*^ = 0.06]. This interaction suggests that the increase in the error rate with magnitude was different for the three arrangements (see Fig. 2c). According to pairwise comparisons, there were significant differences between arrangements only for magnitudes 10 and 12 [*p* < 0.05].

### ERP results

Considering time intervals and locations, different ERP deflections were investigated in a 1000 ms post-stimulus time window. The grand average ERP waveform over a right posterior region of interest (ROI), including P2, P4, PO4 and PO6 electrodes, and the analyzed deflections are presented in Fig. S1 of the Supplementary Material. Although point-by-point analysis did not reveal any significant effect in an early time window, P1 and N1 peak amplitude and latency were nevertheless statistically analyzed by means of ANOVAs with arrangement, magnitude, and hemisphere as repeated measures factors.

#### Early ERP deflections

##### P1 deflection

The repeated measure ANOVA did not reveal any significant main effect or interaction either for peak amplitude or for latency (*ps* > 0.05). For details see Table S2in the Supplementary Material.

##### N1 deflection

Two different ROIs were considered for the N1 component. The first ROI included left- and right-hemispheric occipital and occipito-parietal electrode sites and the second ROI included electrodes over parieto-temporal regions over both hemispheres. According to the repeated measure ANOVA with factors arrangement, magnitude and hemisphere, no significant main effect or interaction was observed (*ps* > 0.05). Detailed statistical results are presented in Tables S3and S4 in the Supplementary Material.

##### N2 deflection

**Figure 3:**
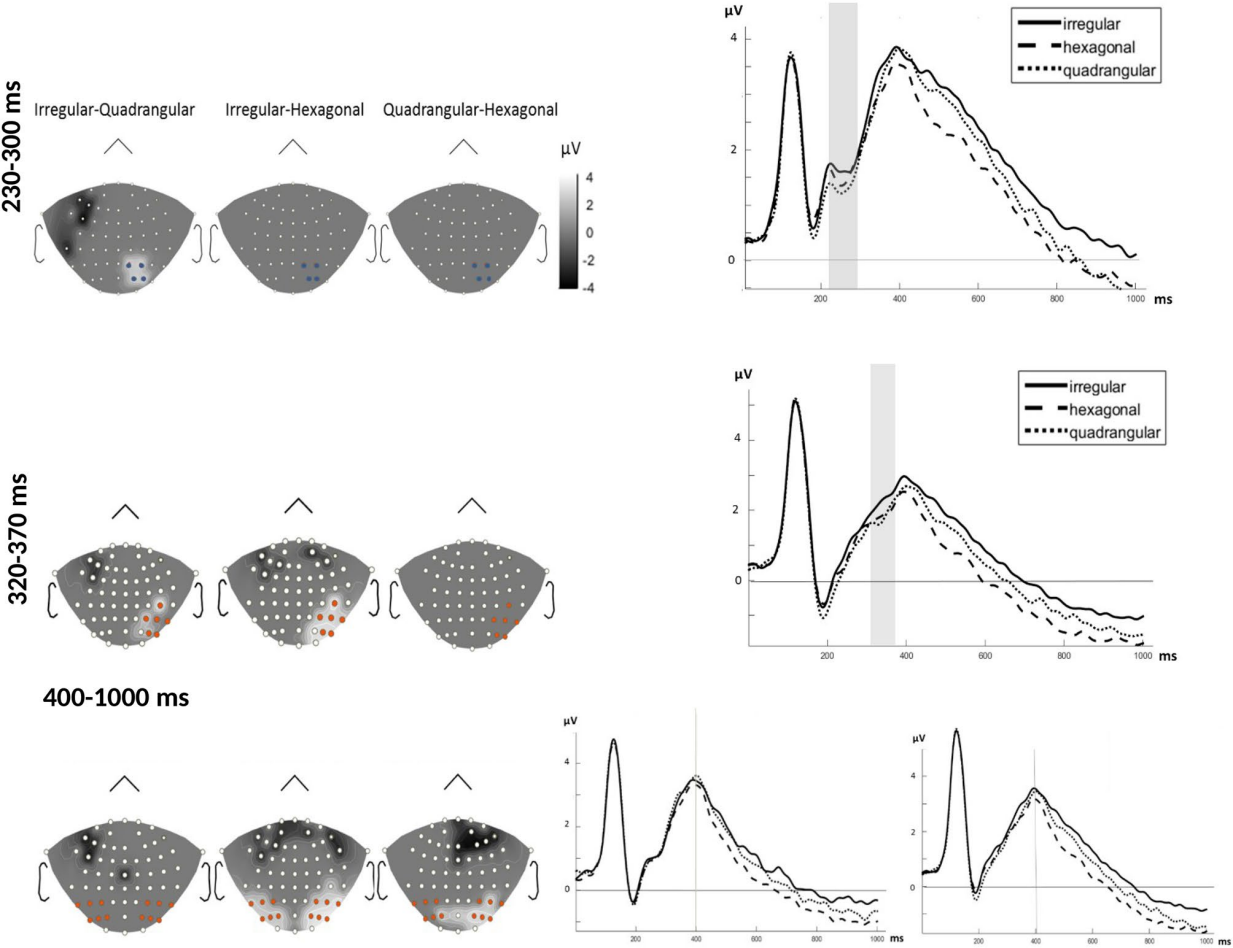
ERP results for different arrangements. Left: Difference topographic plots from the significant time intervals of 230–300 ms (upper panel), 320–370 ms (middle panel) and 400–1000 ms (lower panel) for three arrangement conditions (after FDR correction). Electrodes lied in light area (colored electrodes) entered into the analysis. Right: Time domain ERPs averaged from the electrodes showing the significant arrangement effect. The significant (FDR corrected) time intervals are marked by the shaded area. For lower panel the beginning of the analyzed time window is marked by the vertical line at 400 ms.

According to point-by-point statistics, significant effects of arrangement emerged between 230 and 300 ms at a group of four electrodes over the right parietal and occipito-parietal regions (P2, P4, PO4, and PO6) as shown in Fig. 3. Further analysis by repeated measure ANOVA revealed that the mean amplitude of this component is affected by the arrangement of dots (*ps* < 0.05) but not by their magnitude. Pairwise comparison showed a significantly increased negativity in response to the quadrangular arrangement as compared to the irregular arrangement [*MD* = − 0.338, *p* = 0.004] but not when compared to the hexagonal arrangement [*MD* = − 0.168, *p* = 0.448] (see Fig. 3). The interaction of arrangement and magnitude was not significant (*p* > 0.05). Detailed results of the repeated measure ANOVA are reported in Table 1.

**Table 1:**
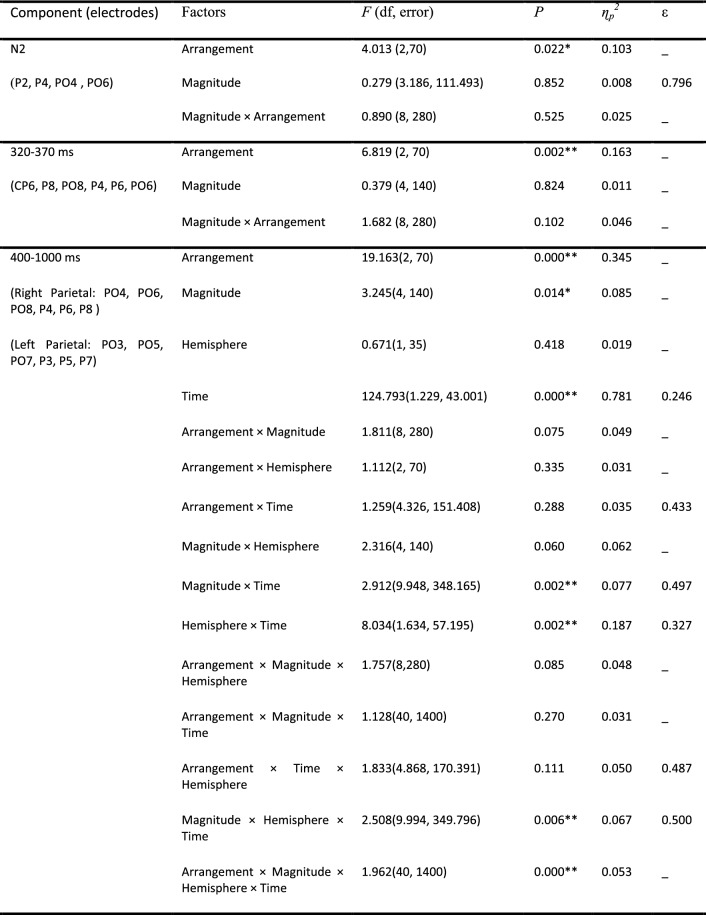
Repeated measure ANOVA results for mean amplitude in significant time intervals.

#### Late ERP deflections

Time-point comparisons indicated a significant time interval between 320 to 370 ms after stimulus onset for electrodes over the right parietal region (CP6, P8, PO8, P4, P6, and PO6), which is displayed in Fig. 3. A repeated measures ANOVA of mean ERP amplitudes for this time interval and region revealed a significant main effect of arrangement (*ps* < 0.01) but not of magnitude. The Magnitude × Arrangement interaction was not significant either (*p* > 0.05). For statistical details see Table 1. Pairwise comparison revealed the arrangement effect to be due to a larger amplitude in irregular sets as compared to regular ones [irregular-hexagonal: *MD* = 0.395, *p* = 0.001; irregular-quadrangular: *MD* = 0.461, *p* = 0.004] (see Fig. 3).

**Figure 4:**
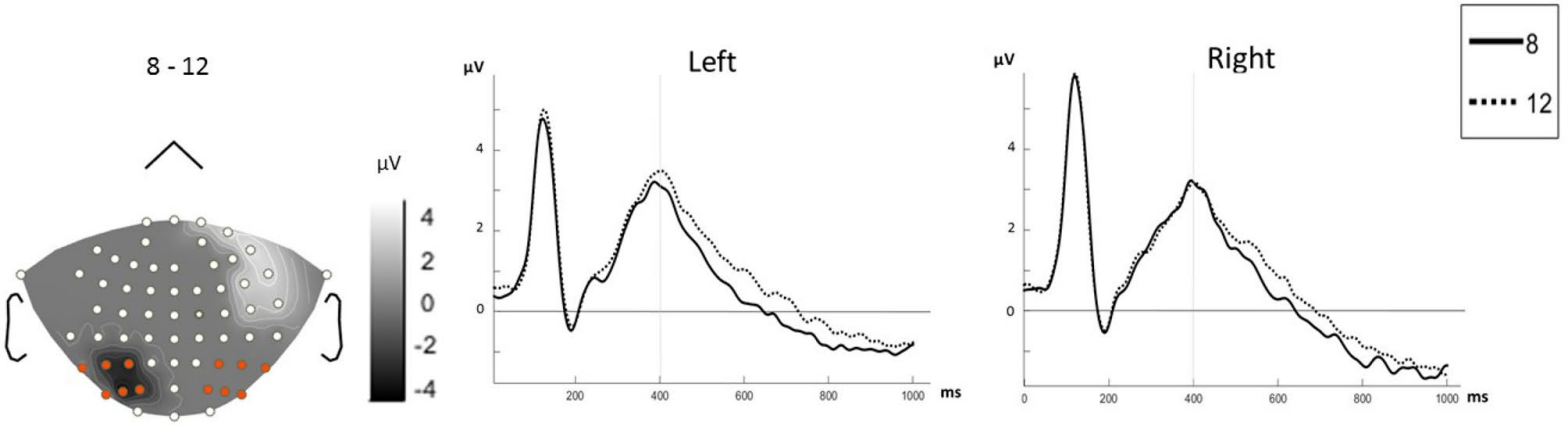
The effect of magnitude on ERP results*.* Left: topographic plot of time interval with significant difference (400–1000 ms) according to *t*-test (after FDR correction) considering magnitude condition. 6 electrodes over right and left parietal areas were entered into the analysis. Right: time domain ERP averaged from electrodes over left parietal (left chart) and right parietal (right chart) showing significant magnitude. The vertical line at 400 ms marks the start of the analyzed time window.

There was a significant effect of arrangement and magnitude between 400 and 1000 ms at electrodes over bilateral parietal regions (see Figs. 3 and 4). The activation strength within these regions was measured over consecutive 100-ms time windows (400–500 ms, 500–600 ms, 600–700 ms, 700–800 ms, 800–900 ms, and 900–1000 ms). The repeated measures ANOVA of mean ERP amplitudes for this time interval and the two regions showed statistically significant main effects of time (defined in terms of the six consecutive 100-ms time windows), magnitude, and arrangement (*p* < 0.001).

**Figure 5:**
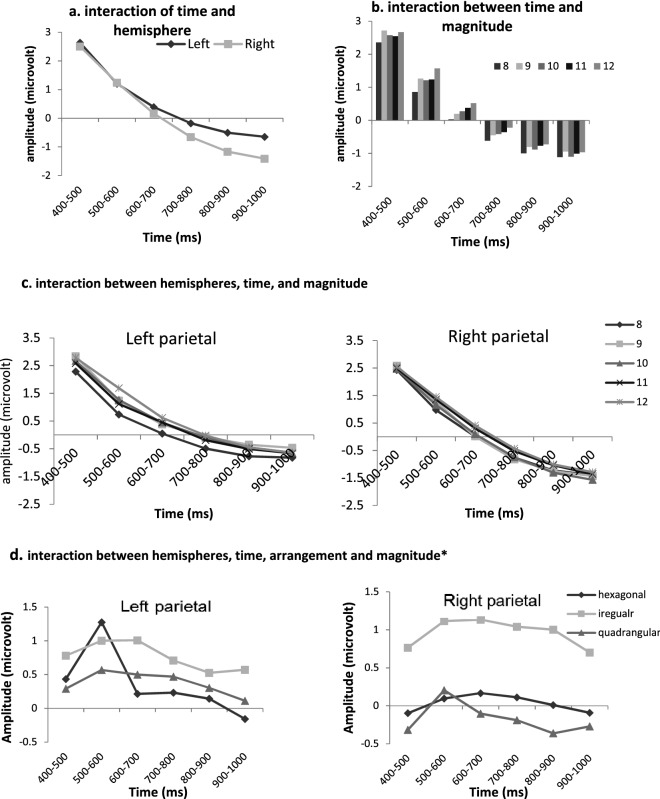
(**a**), (**b**) and (**c**) representation of significant two and three-way interactions observed between time, hemisphere and magnitude factors. (**d**) representation of four-way interaction between time, arrangement and magnitude in two parietals (left: left parietal; right: right parietal). Here magnitude is shown as the difference between smallest (8) and largest (12) magnitude conditions.

**Figure 6:**
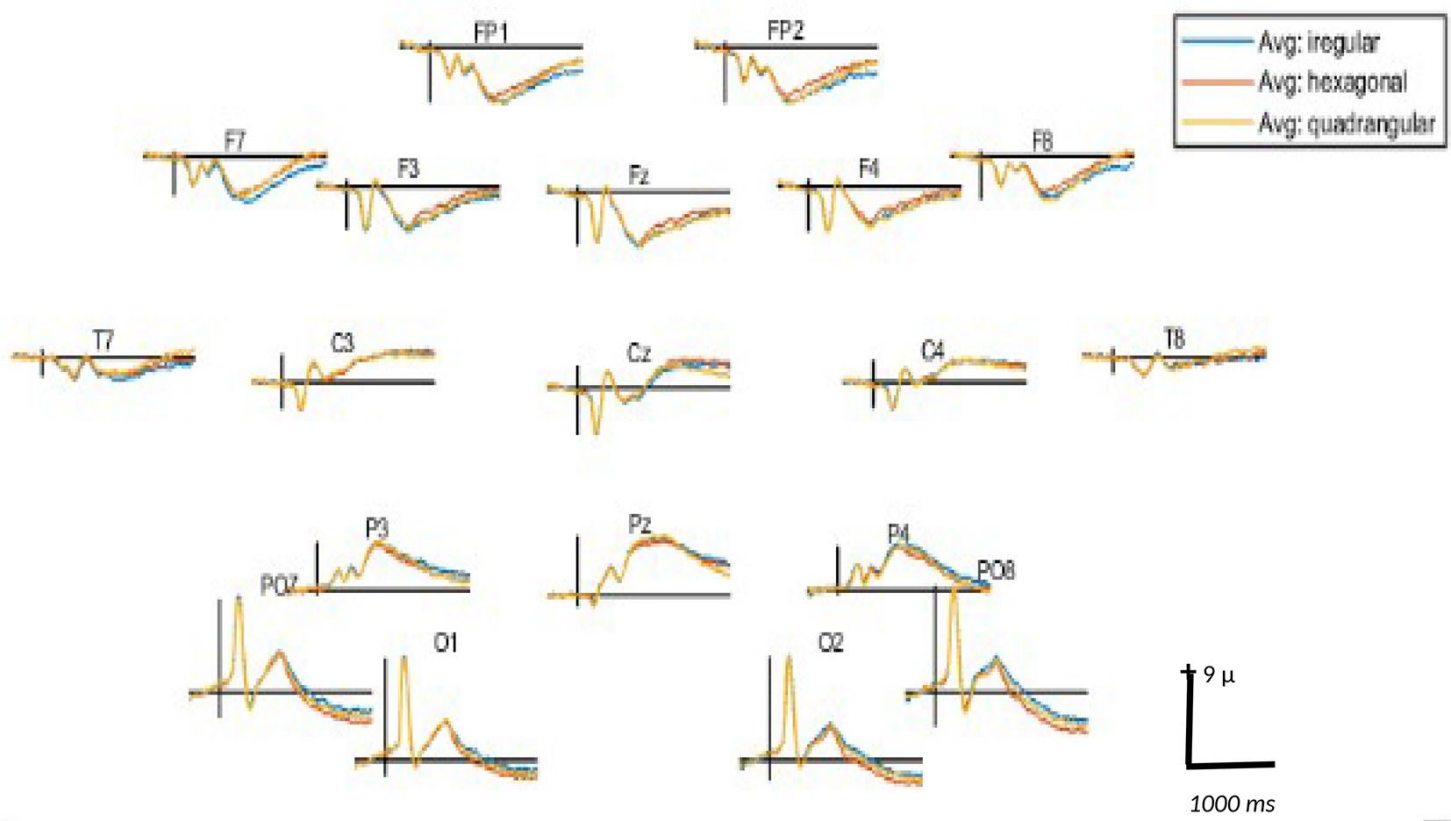
Grand averaged signal in a 19 channel montage for three arrangement displays. Time window: − 200–1000 ms. Positivity is plotted upwards.

**Figure 7:**
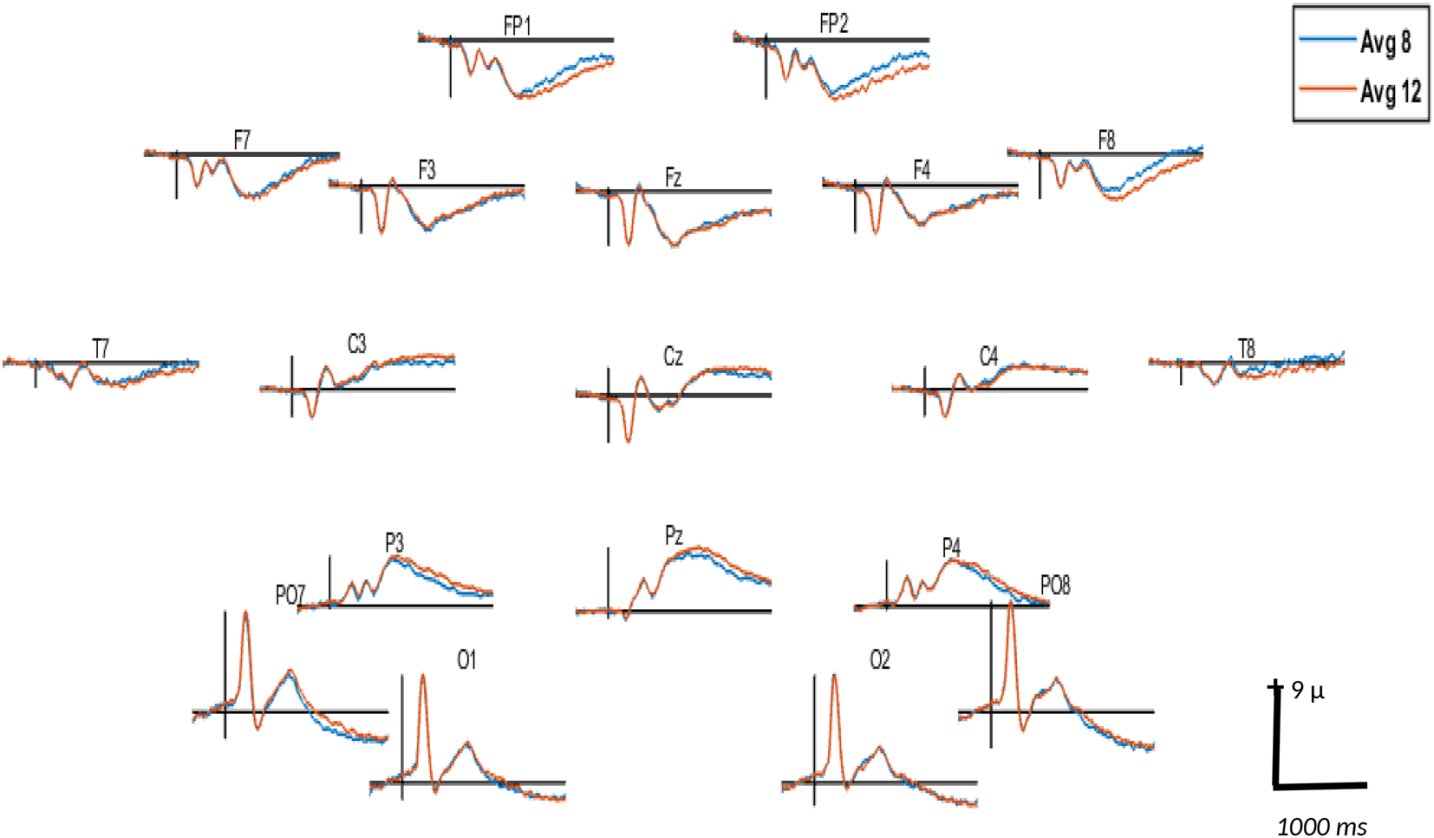
Grand averaged signal in 19 selected channels for 8 and 12 dot displays. Time window: − 200–1000 ms. Positivity is plotted upwards.

The main effect of arrangement was not modulated by other factors (*p* > 0.05). The Time × Hemisphere interaction was significant (*p* < 0.01), indicating that the decrease in activation strength over time for the two hemispheres was larger for the right than the left hemisphere, as corroborated by the slope analysis [*t*(35) = − 3.10, *p* = 0.004] (cf. Fig. 5a). The significant Time × Magnitude interaction (*p* < 0.01) suggests that the amplitude differences between magnitude conditions change over time (see Fig. 5b). Thus, the magnitude effect between 8 dots and the other dot sets was most prominent in the second time interval (500–600 ms) as confirmed by pairwise comparison (*p* < 0.05). The Time × Magnitude × Hemisphere interaction (see Fig. 5c) and the Time × Arrangement × Magnitude × Hemisphere interactions were significant (*p* < 0.01). The latter interaction implies that magnitude processing for different arrangements produced different ERP amplitude patterns over the two hemispheres. That is, irregularity produced a much larger ERP amplitude difference for the magnitude effect (8 vs. 12 dots) over the right hemisphere, whereas this difference between arrangements was less pronounced over the left hemisphere (see Fig. 5d). For irregular arrangements, there was no prominent hemispheric difference for the magnitude effect (8 vs. 12 dots) but this arrangement produced a generally more positive ERP amplitude at electrodes over the two hemispheres than for the two other arrangements. However, for both more regular arrangements, a magnitude effect was only present over the left hemisphere and here most clearly for the initial consecutive time intervals. Statistical results of the repeated measure ANOVA analysis of the late 400–1000-ms time interval are provided in Table 1. For a comprehensive overview of ERP waveforms at 19 selected channels for both factors of arrangement and magnitudes, see Figs. 6 and 7.

### Summary of results

Behavioral results confirmed significantly faster enumeration for more regular and smaller dot sets. The size effect was less obvious for quadrangular arrangements. Enumeration was more error prone for larger sets but the arrangement did not affect counting accuracy. Early components (P1 and N1) were neither affected by arrangement nor magnitude.

The first significant effect of arrangement was observed on mean N2 amplitude (230–300 ms) and subsequently on mean amplitude between 320 and 370 ms. Both of these effects were present over the right parietal region. From 400 to 1000 ms post-stimulus there was a constant effect of arrangement over both parietal regions.

The effect of magnitude was only observed later in the ERP waveform (400–1000 ms), most prominently between 500 and 600 ms post-stimulus, with irregular arrangements producing a particularly clear magnitude effect over the right rather than the left hemisphere.

Source modeling was performed using minimum norm imaging. For details about the source modeling analysis and results see Supplementary Material Figs. S2, S3, S4, S5.

## Discussion

In an exact enumeration task, we compared the brain activity (ERPs) elicited during the enumeration of three different arrangements of dot sets. We investigated (i) how arrangement changes and therefore how illusory contour fluctuations influence the counting process and (ii) which stage(s) of exact enumeration processing are affected by arrangement differences. We measured enumeration speed in different arrangements for which proximity and similarity effects were controlled and the illusory contours were manipulated in the sense of ambiguity (the number of possible chunking strategies). Although it is impossible to absolutely control the effect of proximity and sparsity, we have designed an experiment in which participants were not able to detect predefined groups of dots but rather had to find strategies to chunk them.

### Behavioral findings

Our behavioral results about the speed of enumeration for different arrangements suggest that, in the absence of proximity and similarity, illusory contours still affect enumeration speed in such a way that increasing ambiguity in dot lattices is related to slower enumeration. Imaginary lattice formation leads to grouping and by grouping a small set of items is considered as one object. As a result, subitizing small groups of dots is an important accelerative component of exact enumeration. This makes enumeration a complex serial cognitive process composed of parallel simpler processes.

The general idea that counting is not a one-by-one process is not entirely new. Van Oefflen and Vos (1982) proposed that enumeration is composed of four constituents: (i) a constant amount of time for the perceptual segmentation of a dot set into groups, (ii) the time needed for subitizing within a group, (iii) the time to compute the summation of groups, and (iv) a constant amount of time for response execution. Therefore, response latency is a function of both the total number of dots and the number of groups as well as the duration for the grouping of items. However, Van Oefflen and Vos assumed a constant amount of time for grouping. In their experiment grouping was conducted by proximity; therefore, their account does not explain the influence of the illusory contours and arrangement seen in the current study. Furthermore, it does not explain the present finding of an interaction between arrangement and magnitude in regard to enumeration speed.

Although increasing the magnitude slows down the enumeration process in general, this slowing effect is more pronounced for the most ambiguous irregular arrangement. However, the slope analysis revealed a strong linear decrease of enumeration speed with magnitude for all arrangements, even for the regular quadrangular arrangement, which had the gentlest slope. This finding is inconsistent with the idea that counting by grouping arrays is unaffected by the size of the set as suggested by Mandler and Shebo (1982) and Starkey and McCandliss (2014). Here it is worth mentioning that besides arrangement and regularity, the applied counting strategy also affects enumeration speed. As indicated by the behavioral results (Fig. 2a), the slope of the RT increase is not constant but changes over odd and even numbers. Specifically, for those numbers for which chunking and subitizing was not the only counting solution (eg., 9, 11), RT increased with a steeper slope. This means that participants need more time to calculate the numerosity. For instance, if chunking sets in rectangles, 11 will be calculated as (3 × 3) + 2 whereas 12 will be calculated as (3 × 4). These slope changes are more obvious in the case of irregular and hexagonal arrangements which supports our claim about different strategies of enumeration for different arrangements. For instance, if participants chunk rectangles and then count the rest, counting 9 or 11 dots should be relatively slower than 8 or 12 dots, especially if we face ambiguity in chunking strategies.

An additional finding was that, in contrast to RT, accuracy of enumeration was not affected by arrangement, while magnitude seems to be a determining factor of accuracy. This finding is in line with previous research indicating that differences between enumerating small and large numbers of items are manifest not only in latency functions, but in error rates and confidence ratings as well^38,39^. Finally, the interaction of arrangement and magnitude in error rates implies that although regularity in the arrangement does not guarantee the correctness of responses, it does modulate the effect of magnitude on response accuracy.

### ERP findings

As mentioned in the introduction section, for ERP findings, we considered two separate time phases including an early (before 300 ms post-stimulus) and a late processing stage (after 300 ms post-stimulus). This segmentation approximately corresponds to the distinction between more sensory-related and cognition-related processes, with the latter first reflected in the ERP waveform by the P300 component. The P300 is viewed as an indicator of attention, cognitive processing performance, and meaningfulness associated with the stimulus^40,41^.

#### Early-stage processing

With regard to early-stage processing, first, it is important to note that the amplitude of visual P1 and N1 components (indicators of early visual processing) was not influenced by arrangement and magnitude. This null finding is in accordance with the view that early sensory ERP components are mainly affected by more salient visual parameters such as proximity, size, and sparsity^42,43^. Since these factors were controlled in our task, this result is not very surprising. However, this finding is in contrast to some recent research findings which indicate that numerosity modulates early components during visual and number processing^44–46^. A closer look at these studies suggests that they differed in stimulus range, tasks, and responses as compared to our study. For instance, Hyde & Spelke (2009) adapted subjects to small and large sets, which were obviously different in magnitude (1, 2, or 3 vs 8, 16, or 24 dots). Therefore, sparsity and density were not controlled in their task.

Crucially, the present ERP results suggest that arrangement, as a visual characteristic of stimuli, reliably changes at a relatively early phase of the enumeration process at about 230–300 ms post-stimulus. According to our earlier explanation, this time interval is attributed to the late-global high-level mechanism of illusory contour perception which probably reflects feedback mechanisms arising from visual areas of the brain. In this so-called high level mechanism, the global shape of the structure (in our case arrangement of the dots) is represented^29^. We may infer that the strategy of chunking is selected according to the general form of the arrangement which is perceived at this stage and results in later differences in enumeration process.

More specifically, the effect of arrangement was first observed in the N2 (230–300 ms), which was apparent in the ERP waveform as a negative deflection that peaked at about 270 ms post-stimulus. The N2 is known to be modulated by different types of stimulus features such as color^47^, position^48^ and shape^49,50^. In the light of the present N2 amplitude modulations, it seems that the N2 is also affected by our arrangement manipulations. As explained earlier, arrangement changes induce different grouping strategies due to their effect on visual contours. Thus, we suggest that the differentiation between dot lattices is realized during the N2 time interval. Soltesz and Szűcs (2014) pointed to a similar ERP negativity as being sensitive to unattended changes in stimulus features that reflect automatic and unconscious detections of alterations in the environment. Therefore, although these features do not influence very early components, we infer that illusory dot lattices and grouping should be considered as basic features of visual stimuli which are processed unintentionally and automatically. It is worth noting that the N2 amplitude differences between experimental conditions were only observed over the right occipito-parietal region. Current source density analysis did not show a significant difference between the sources for the three different arrangements (see the Supplementary Material).

The occipito-parietal area is typically activated during focused visual attention^51^ and grouping by the Kanizsa effect^52^. Most studies mention bilateral activation of the occipitio-parietal area in grouping tasks^24,25,42^. Our results confirm that the occipitio-parietal region is also activated in the processes related to the “differentiation of grouping strategies” caused by different arrangements. In accordance with behavioral results, the largest N2 amplitude difference emerged between irregular and quadrangular arrangements, suggesting that irregularity and ambiguity in grouping strategies increases the amplitude of this ERP component (see Fig. 3). Since contour perception is attentionally more demanding^53^ due to its lower salience, we can interpret this N2 effect in terms of an increase in attentional processing for more ambiguous sets. Considering the fact that grouping is a technique for getting and keeping information in short term memory, its modulations will affect encoding and other related processes.

#### Late-stage processing:

It is interesting to mention that about 50–100 ms after the N2 effect, the ERP waveform indicated a reliable difference between the irregular and the two regular (quadrangular and hexagonal) arrangements at 320–370 ms post-stimulus. One explanation for this difference is that the differentiation between regularity and irregularity is taking place at this time interval. On the other hand, it might be due to the differences in later processing stages such as encoding and storing chunked arrays. Here, again, no significant difference was shown between current source density maps (see the Supplementary Material).

To summarize, the ERP effect of grouping by different Kanizsa edges occurs much later than for other visual factors such as proximity, and like other configurational effects it is predominantly observed over the right hemisphere and extends to later components after the N2. Even without referring to magnitude effects yet, this shows that the visual aspects of exact enumeration examined in the present study differ from the visual principles studied before^11^, which may explain diverging results.

We also investigated higher-level processing of enumeration at a time window of 400 to 1000 ms post-stimulus in the ERP waveform. Since the fastest average response latency was observed in 8 dot sets and the minimum reaction time was around 1500 ms, we assume that in this time interval, mainly processes related to effortful counting rather than later response execution are observed (see also^54,55^). Considering the effect of arrangement, again the clearest ERP amplitude differences between conditions were observed over right parietal regions. However, the source of this difference between two more ambiguous arrangements was identified over the right fronto-temporal area (see Fig. S4 in the Supplementary Material). Therefore, we might assume that this area participates in distinguishing the arrangement during a cognitive task.

Magnitude caused a larger activity difference over the left parietal area, which might imply that the ERP effect of magnitude is observable over this region. However, source estimations showed the source of this difference to be located in the central parietal and right frontal cortex (see Fig. S5 in the Supplementary Material). This finding indicates the greater attentional modulation and shifts of attention during the enumeration of larger sets. The role of the right frontal lobe in counting has also been mentioned by previous studies^56–58^. Nieder et al., (2004) reported that during numerical judgment, information is conveyed from the posterior parietal area to the prefrontal area. Since we did not find any interaction between arrangement and magnitude during this time interval, we tentatively suggest that participants used the same numerosity processing during counting in all three types of arrangements.

Our ERP findings also showed that the magnitude was processed around 500–600 ms post-stimulus (see Fig. 5b). This is consistent with the results reported by other studies (e.g.^59^) observing magnitude effects on late ERP components. Such studies suggested that behavioral differences in response latency caused by magnitude are not due to early visual processing but result from later cognitive processing. The stronger right parietal ERP activity during late processing indicates lateralized brain activity during conceptual number processing taking place at higher levels of the exact enumeration process^60^. We can interpret the four-way interaction (see Fig. 5d), as possibly suggesting that regularity helps in the computation of the small and large numbers in the same manner at later phases of the enumeration process, when the visual addition is almost done and activity is likely related more to verbally-mediated processes. However, increased ERP activity in bilateral parietal regions in response to irregularity might represent more widespread brain activity due to simultaneous visual and verbal addition—non-symbolic and symbolic processing, respectively—during enumeration of these arrangements. Therefore, for irregular arrangements, magnitude is more important. This is in accordance with our behavioral results in which enumeration in irregular sets was shown to be more affected by set size, because sequential addition of chunking subitized groups of dots becomes much harder when the dots cannot be easily grouped.

### Limitations of this study

We have to acknowledge that the present ERP analysis is potentially limited by the relatively small number of trials per condition (30 trials). However, we calculated ERP amplitude for ROIs for which signals from multiple electrodes were averaged. By doing so, the signal to noise ratio (SNR) substantially increases. On the other hand, considering the 3 × 5 design, we had 30 × 5 = 150 (for arrangement) and 30 × 3 = 90 (for magnitude) trials contribute to the condition means for the main effects, which constitute the main part of the manuscript. However, we acknowledge the fact that for 3 × 5 interactions we have 30 trials per cell, which constitutes a low number. Together, whereas we occasionally interpreted differences between conditions with 30 trials only, with regard to our main conclusions, our inferences are based on analyses to which many more trials contributed. In other cases, we computed linear trends, which constitutes a much more restricted type of analysis than an ANOVA, where any possible difference is computed.

A second limitation is that when surface area and dot size is kept constant, there is necessarily mathematically a direct relation between numerosity and density of the dots (more dots in the same area means higher density). Therefore there is a trade-off between controlling density and area size and it is not possible to control both of them simultaneously. However we should consider the fact that since we had a small range of number magnitudes (8 to12) and the distance between the minimum and maximum magnitudes is only 4 numbers/dots (note that in many numerosity task, variation is between 3–4 and 25–100 dots, e.g. 6–30 times more dots in the most frequent number, here the maximal difference is 1.5 as many). Moreover, when designing our slides we checked whether there is a relation between surface area and magnitude. There was no significant relation [F(4, 70) = 1.85, *p* > 0.05]. So neither the proximity effect (which is of course excluded from the stimuli by equal distances) and nor the early components take effect of magnitude changes*.* Therefore, we do not think, that our results were affected by these variations to a significant extent.

### Summary and a proposal for a model framework to explain the data

Overall, our results suggest that promoting grouping probability by regular lattices has a crucial effect on exact enumeration speed and chunking as an automatic process (< 300 ms) is a prerequisite for more fluent counting.

As regards magnitude processing, we observed that there was no perceptual cue for magnitude in early ERP components. Magnitude processing, however, was evident at much later stages of cognitive processing due to differences in attentional modulations. The late activation of the left parietal region presumably reflects counting-related mathematical operations and verbally mediated addition. In contrast the right parietal region may be mostly devoted to visuospatial processing and later conceptual processes of exact numerosity, like controlling chunking, especially in slower and more irregular arrangements. Our findings suggest lateralized brain activity during conceptual number processing to take place at higher levels of the exact enumeration including more complex cognitive processing such as mathematical calculations.

Therefore, we propose an Account for Sequential Subitizing Addition (ASSA), which suggests that adult counting is not necessarily a sequential shifting over items one-by-one but consists of sequential subitizing and subsequent addition of subitized groups. However, sequential subitizing is strongly affected by arrangement of the sets (i.e., regular vs irregular). Therefore, we suggest a revised and extended model, which is based on the previous work of Van Oefflen and Vos (1982).

(i) Detect Kanizsa edges, which can be used for perceptual segmentation
(ii) After perceptual segmentation chunk the dots into small groups with N < 4 (i.e., in the subitizing range). This is harder for more irregular arrangements
(iii) Read out the size of each group by subitizing
(iv) Memorize this size as a symbolic number
(v) (Verbal) computation of symbolic number words
(vi) In the case of unclear perceptual segmentation and chunking, visuo-spatial control of the chunked dot patterns in late stages, especially for irregular patterns that were hard to chunk
(vii) Response execution

Therefore, considering the role of arrangement and grouping in enumeration, if we wish to teach fast and efficient adult-like counting to children, we may not only teach them one-by-one counting and shifts of attention, but also grouping and subitizing of countable objects.

## Materials and Methods:

### Participants

A total of 37 university students (14 males, mean age: 24.21 ± 2.15) participated in this study. All participants were right-handed, had normal or corrected-to-normal vision, and no history of psychiatric or neurological disorders. Written informed consent was obtained from all participants. All procedures of the study were in accordance with the latest revision of the Declaration of Helsinki and were approved by the ethics committee at the University of Tabriz.

### Stimulus

Three different arrangements were designed: a regular quadrangular arrangement, a regular hexagonal arrangement, and an irregular arrangement. Similarly to work by Gebuis and Reynvoet (2011, 2012)^9,61,62^ and Salti et al., (2017)^63^, we controlled our stimuli for different visual properties such as area, size, distances (proximity), and arrangement using a self-written MATLAB script. The quadrangular arrangement embodied lattices in the shape of a quadrangle. Although the rectangular lattice is the most stable according to Kubovy et al., (1998), the relatively stable quadrangular lattice was selected as its equidistant arrangement of dots minimized the potential influence of proximity (see Fig. 1d). The hexagonal arrangement, a more ambiguous regular arrangement^64^, consisted of parallel rows of dots at equal distance which were positioned 60 degrees from one another (see Fig. 1c). The irregular arrangement consisted of irregularly scattered dots (see Fig. 1b). The distance between individual dots was kept approximately equal in order to minimize the chance of grouping by proximity (see below).

The stimuli contained 75 dot patterns which included 8 to 12 dots in three different arrangements. Therefore, there were 15 conditions (5 magnitudes in 3 arrangements). Black dots were scattered at the center of a white circle with a 14 cm diameter (visual angle=5.72°), their distances were kept as equal as possible while sparsity was controlled to minimize any proximity effect. Sparsity, defined as the summed area of the dots, was kept constant (27.2±1.2 cm^2^, means subtending approximately 2.45° of visual angle) as also reflected by the fact that the ANOVA showed that this area did not significantly differ across the different magnitudes [*F*(4, 70)=1.85, *p*>.05]. This result made it rather unlikely that participants judge numerosity simply on the basis of the cumulative surface area of the sets. To make sure of this, before starting the main experiment, slides were presented to a group of 10 participants and their enumeration speed was calculated. Those dot patterns which caused unusually fast responses (detected as outliers) or for which participants reported the formation of a symbolic pattern were replaced with other dot patterns. For an overview of typical 3×5 stimulus arrays see Fig. S6 in The Supplementary Material.

Using Eevoke 3.1 software (ANT Neuro, Netherlands), we designed five different stimuli for each number of dots (8–12) in each arrangement category (e.g., five slides for irregular arrangements of eight dots), resulting in 75 different slides.

### Apparatus

**Figure 8:**
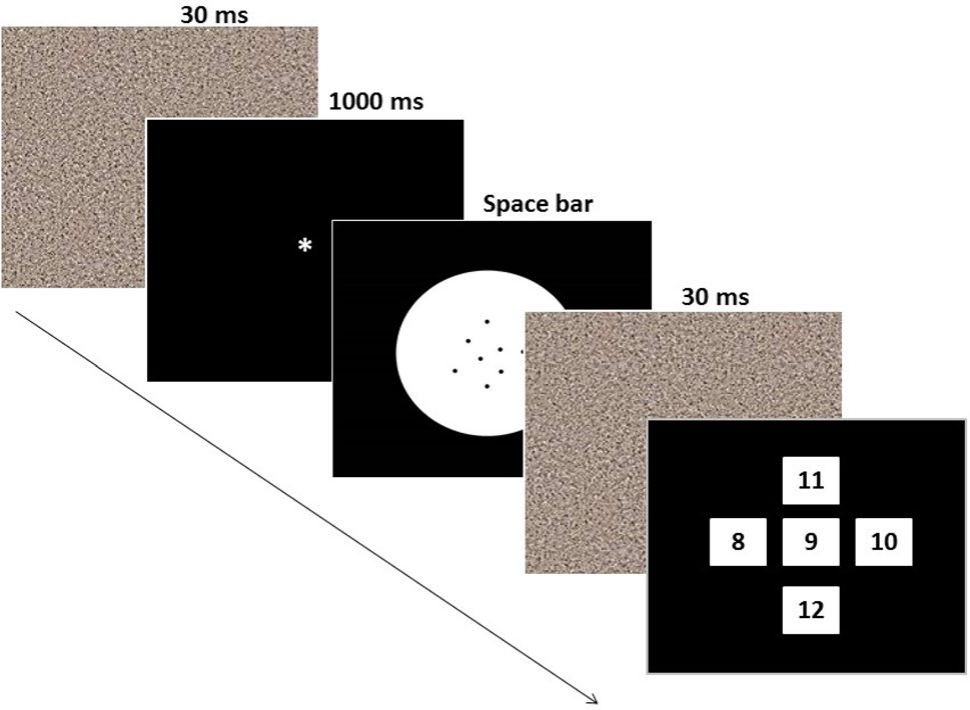
The task diagram.

Participants were tested individually. The stimuli were presented at the center of a 23-inch computer monitor at a viewing distance of approximately 1.4 m, subtending approximately 3˚ × 3˚ of visual angle. Each trial consisted of three successive phases. First, there was a yellow fixation cross at the center of the black screen for 1000 ms. It was followed by the presentation of a dot pattern in a white 14-cm diameter circle that was centered on the screen. Participants were not informed about the research objectives. They were asked to count the dots as quickly as possible and to press the space bar with their left hand immediately after they completed counting. Then, the response screen was displayed from which participants were to choose the correct answer out of the five given alternatives, and to enter their response by pressing the spatially corresponding key on the number keyboard using their right hand. The presentation of dot sets and response screen was terminated by the participants’ key press response (see Fig. 8). There was a time limitation of 8 s for the counting stage, however, participants were not aware of this time limit. The reason was to prevent probable time estimation processes and temporal judgments, which could affect ERP deflections^65,66^. No feedback was given as to the appropriateness of responses. To prevent afterimage formation, in each trial a 30 ms texture masking was applied before the fixation point and after the dot set slide (see^67,68^). The stimulus set consisting of 75 slides was repeatedly presented in six blocks, resulting in a total of 450 trials. In other words, each condition contained 30 trials. Conditions were presented in a random order within each block. Each participant practiced the task in a block of 30 trials before the experiment.

### Electrophysiological recordings and preprocessing

Throughout the experiment, electrical activity of the brain was recorded via an ANT-64 channel electroencephalography (EEG) amplifier (ANT Company) from 64 electrodes arranged according to the 10–10 system^69,70^ with an online average reference. The impedance was kept below 10 kΩ. Data were digitized at 500 Hz and an online band-pass filter (0.016 to 100 Hz) using ANT ASA-lab software was applied.

Preprocessing and data analysis was performed with Brainstorm^71^, which is documented and freely available for download online under the GNU general public license (http://neuroimage.usc.edu/brainstorm). EEG signals were filtered offline, using a band-pass filter of 0.1–30 Hz. Bad EEG segments (those exceeding ± 100 μV in any channel) and eye-movements were rejected and eye-blink artifacts were corrected using an Independent Component Analysis approach^72^. Visual inspection was carried out after the rejection to assure quality of the data. Data from one subject with less than 65% of trials remaining after artifact rejection were excluded from further analysis. In the remaining data set, a total of 9.6% of the trials were excluded (4.3% due to wrong responses and 5.3% due to artifacts).

Epochs from -200 to 1000 ms relative to stimulus onset (at t = 0 ms) were extracted and baseline-corrected to a pre-stimulus interval of -200 to 0 ms.

### Behavioral data analysis

The reaction times and number keyboard responses were recorded using the Eevoke 3.1 software. Trials with an incorrect response were excluded from data analysis and the error rate was determined. Since error rate was not distributed normally, it was transformed by taking the arcsine of the square root of the error rate. RT was defined as the time between the onset of the dot set presentation and the space bar response. 2.6% of RTs that were 1.5 times larger than the upper interquartile range and 1.5 times smaller than the lower interquartile range were considered outliers and removed. This way we tried to significantly decrease the chance of considering those trials in which counting likely did not take place and participants guessed the numerosity. Data were analyzed using repeated measurement ANOVAs on within-subject factors (arrangement and magnitude). Pairwise comparisons were used post hoc and *p*-values were adjusted by the false discovery rate (FDR) correction for multiple comparisons^73^. The Greenhouse–Geisser correction was applied to degrees of freedom whenever the sphericity assumption was violated. All statistical analyses were conducted using SPSS version 21 and *p*-values < 0.05 were considered statistically significant.

### ERP measurement

For artifact-free trials, the EEG signal at each electrode was averaged separately for each participant and each condition. These resulting average ERP waveforms were used in the statistical analysis as detailed below. Grand averaging was performed separately across arrangement and magnitude, resulting in three grand averages for arrangement and five grand averages for magnitude. These grand averages were used for visualization of the main effects of arrangement and magnitude on ERP waveforms.

Due to the exploratory nature of the study, our statistical analysis consisted of two main steps: the first step was defining the ROIs and time intervals of interest which were affected by our independent variables (arrangement and magnitude). The second step was the statistical analysis to investigate how these ROIs and time intervals were affected by our factors.

While the most common approach to define the ROIs and time points is the visual inspection of the grand average, an alternative method is to use a point-by-point permutation t-test which has been used for EEG data^50,74,75^ and other types of signals such as functional near-infrared spectroscopy^76^. It was an appropriate approach for the current study because the grand average was not so informative and would lead to type II error (false negative).Therefore in the first step, we conducted point-by-point permutation t-test (with time points of 2 ms and in the 0–1000 ms post stimulus time window) between different arrangements (while collapsing data across different magnitudes) with 1000 permutations to find the first candidate ROIs and time points for further analysis. For instance, we compared irregular sets (including all magnitudes) with regular sets (including all magnitudes). We repeated the same procedure between different magnitudes (and here collapsed data across different arrangements). For instance, we compared 12 dots (including all arrangements) with magnitude 8 (including all arrangements). We applied some inclusion criteria consistent with previous studies^77,78^ as follows: Whenever a significant effect (*p* < 0.05) was observed in more than two neighboring electrodes with at least 5 consecutive data points (10 ms) those electrodes were considered as a cluster and the mean amplitude over this time interval and cluster was extracted to be included in further analysis. We considered the fact that early components (less than 300 ms) are not expanded in time and usually take place in a short time interval. To avoid type I error (false positive), we corrected alpha level for multiple testing using False Discovery Rate (FDR) method in Brainstorm software.

After finding our ROIs and time intervals, in the second step, for statistical comparison of peak/mean amplitude of ERP components, data from 15 conditions were exported to MATLAB software and analyzed by repeated measures ANOVAs with the factors arrangement, magnitude, and hemisphere (whenever a significant effect was observed over both hemispheres in the first analysis step above; otherwise, the factor hemisphere was dropped, as for the analysis of the N2 and the 320–370-ms time interval). Since the ERP waveform showed a large sustained deflection from 400 to 1000 ms post stimulus, this time window was divided into consecutive windows of 100 ms; a similar method was used by other researchers facing such sustained ERP deflections^51,76,77^ Again, clusters were selected according to the above mentioned permutation *t*-tests and in each cluster mean amplitudes during these consecutive 100-ms time windows were calculated and entered into the statistical analysis. Therefore, for the late 400–1000-ms time interval, time was considered as an additional factor, a similar method is used by other researchers facing such sustained ERP deflections^54,79,80^ Again, clusters were selected according to the above mentioned permutation *t*-tests and in each cluster, mean amplitudes during these consecutive 100-ms time windows were calculated and entered into the statistical analysis. Therefore, for this late 400–1000 ms time interval, time was considered as an additional factor.

### Calculating P1 and N1:

Since the permutation test showed no significant difference in early visual ERP deflections (P1 and N1 components), these components were identified based on topography, polarity, and latency parameters. P1 was considered as the most positive peak in the 80–180 ms post-stimulus interval over left and right occipital and occipito-parietal electrode sites (i.e., O1, PO3, PO5, and PO7 on the left side and O2, PO4, PO6, and PO8 on the right side), consistent with previous reports ^81,82^. Separately for each hemisphere, these electrodes were clustered and P1 peak amplitude and latency was determined for the two clusters. The N1 deflection was defined as the most negative deflection in the 160–250 ms post-stimulus interval and was most pronounced over left and right parieto-temporal and inferior parietal areas (i.e., T7, TP7, and P7 on the left side and T8, TP8, and P8 on the right side). By clustering these electrodes on each hemisphere, N1 peak latency was investigated for two different scalp regions: first, for occipito-parietal electrode sites (O1, PO3, PO5, and PO7 on the left side and O2, PO4, PO6, and PO8 on the right side) in line with previous studies^50,74^, and second, over the area for which N1 amplitude was maximally negative. For later ERP deflections, only their amplitude was analyzed.

The Greenhouse–Geisser correction was applied to degrees of freedom whenever the sphericity assumption was violated, however, uncorrected degrees of freedom are reported. Pairwise comparisons were used post hoc and *p*-values were adjusted by the FDR correction for multiple comparisons. For source estimation, minimum norm imaging was applied to estimate the cortical current source densities (CSD).

## Supplementary Information

Supplementary Information.

## Data Availability

The datasets generated in the current study are available from the corresponding author by reasonable request.
