# Supplementary material for: The complexity of simple counting: ERP findings reveal early perceptual and late numerical processes in different arrangements
Source: Sci Rep. 2022 Apr 26;12:6763. doi: 10.1038/s41598-022-10206-y (PMC9042952; doi:10.1038/s41598-022-10206-y)
Supplement: Supplementary file 1 — Supplementary Information. [file 41598_2022_10206_MOESM1_ESM.docx]

**Supplementary Material**

The complexity of simple counting: ERP findings reveal early perceptual and late numerical processes in different arrangements

Shadi Akbari

Cognitive Neuroscience Lab, Department of Psychology, University of Tabriz, Tabriz, Iran

Mojtaba Soltanlou

School of Psychology, University of Surrey, Guildford, UK

Department of Psychology, University of Tuebingen, Tuebingen, Germany

Hassan Sabourimoghaddam

Cognitive Neuroscience Lab, Department of Psychology, University of Tabriz, Tabriz, Iran

Hans-Christoph Nuerk*

Department of Psychology, University of Tuebingen, Tuebingen, Germany

Leibniz-Institut für Wissensmedien, Tuebingen, Germany

LEAD Graduate School & Research Network, University of Tuebingen, Tuebingen, Germany

Hartmut Leuthold

Department of Psychology, University of Tuebingen, Tuebingen, Germany

*Correspondence

**1. Statistical results**

**Table S1.** Descriptive statistics of RTs (ms) and number of errors in all 15 conditions (N=37).

|  | |  | |  |  |  |  |  |
| --- | --- | --- | --- | --- | --- | --- | --- | --- |
|  | Minimum | | Maximum | | Mean | Std. | Average of errors | Std. of errors |
| Ireg.8 | 1637.62 | | 3432.96 | | 2555.6258 | 411.14060 | 0.43 | 0.555 |
| Hex.8 | 1497.17 | | 3541.33 | | 2295.0881 | 443.99979 | 0.70 | 1.175 |
| Quad.8 | 1528.87 | | 3174.60 | | 2288.8865 | 403.48833 | 0.54 | 0.836 |
| Ireg.9 | 2038.13 | | 4348.58 | | 3054.3355 | 549.87341 | 0.78 | 1.250 |
| Hex.9 | 1925.73 | | 4058.63 | | 2789.2112 | 458.14843 | 1.14 | 1.273 |
| Quad.9 | 1480.45 | | 3594.45 | | 2406.0909 | 432.30997 | 0.86 | 1.294 |
| Ireg.10 | 2038.31 | | 5021.70 | | 3490.5250 | 675.65494 | 1.41 | 1.518 |
| Hex.10 | 1965.25 | | 4142.74 | | 3054.4745 | 551.08713 | 1.03 | 1.067 |
| Quad.10 | 1807.97 | | 4063.68 | | 2638.5766 | 568.04703 | 0.78 | 1.228 |
| Ireg.11 | 2456.10 | | 5395.23 | | 3956.4215 | 739.35663 | 1.76 | 2.722 |
| Hex.11 | 2238.87 | | 5677.57 | | 3446.8046 | 692.06755 | 1.73 | 1.644 |
| Quad.11 | 1905.90 | | 4724.43 | | 2895.3304 | 595.18186 | 1.57 | 1.741 |
| Ireg.12 | 2861.82 | | 6154.50 | | 4187.5037 | 744.11018 | 1.54 | 1.909 |
| Hex.12 | 2463.90 | | 5430.21 | | 3859.5151 | 704.25947 | 1.16 | 1.692 |
| Quad.12 | 1995.53 | | 5246.59 | | 3183.8631 | 716.74574 | 1.62 | 1.534 |
| Age | 21 | | 30 | | 24.21 | 2.158 |  |  |
|  |  | |  | |  |  |  |  |

Abbreviations: Ireg., irregular arrangement; Hex., regular hexagonal arrangement; Quad., regular quadrangular arrangement.

**Table S2.** Repeated measure ANOVA results for peak amplitude and latency of P1 component over two hemispheres (electrodes: O1, PO3, PO5, PO7 on the left side and O2, PO4, PO6 and PO8 on the right side).

| Factors |  | *F* (df, error df) | *P* | *ɳ_p_^2^* | ε |
| --- | --- | --- | --- | --- | --- |
| Amplitude | Arrangement | 0.097(2,70) | 0.908 | 0.003 | _ |
|  | Magnitude | 1.069(2.90,101.59) | 0.365 | 0.030 | 0.798 |
|  | Hemisphere | 2.61(1, 35) | 0.115 | 0.069 | _ |
|  | Magnitude × Arrangement | 0.870(8, 280) | 0.542 | 0.024 | _ |
|  | Magnitude × Hemisphere | 1.766(4, 140) | 0.139 | 0.048 | _ |
|  | Arrangement × Hemisphere | 2.192(2, 70) | 0.119 | 0.059 | _ |
|  | Magnitude × Arrangement × Hemisphere | 1.396(5.33, 186.70) | 0.224 | 0.038 | 0.801 |
| Latency | Arrangement | 0.025 (2,70) | 0.975 | 0.001 | _ |
|  | Magnitude | 2.650(3.30, 115.49) | 0.470 | 0.070 | _ |
|  | Hemisphere | 0.755(1, 35) | 0.391 | 0.021 | 0.921 |
|  | Magnitude × Arrangement | 1.085(5.29, 185.41) | 0.371 | 0.030 | 0.794 |
|  | Magnitude × Hemisphere | 1.894(4, 140) | 0.115 | 0.051 | _ |
|  | Arrangement × Hemisphere | 0.289(2, 70) | 0.750 | 0.008 | _ |
|  | Magnitude × Arrangement × Hemisphere | 0.710(4.73, 165.740) | 0.609 | 0.020 | 0.696 |

**Table S3.** Repeated measure ANOVA results for peak amplitude and latency of N1 component over occipital and occipito-parietal electrodes (electrodes: O1, PO3, PO5, PO7 on the left side and O2, PO4, PO6 and PO8 on the right side)

|  | Factors | *F* (df, error) | *P* | *ɳ_p_^2^* | ε |
| --- | --- | --- | --- | --- | --- |
| Amplitude | Arrangement | 0.318(2, 70) | 0.729 | 0.009 | _ |
|  | Magnitude | 0.305(4, 140) | 0.874 | 0.009 | _ |
|  | Hemisphere | 0.315(1, 35) | 0.578 | 0.009 | _ |
|  | Magnitude × Arrangement | 0.585(8, 280) | 0.790 | 0.016 | _ |
|  | Magnitude × Hemisphere | 1.194(3.066, 107.307) | 0.316 | 0.033 | 0.848 |
|  | Arrangement × Hemisphere | 1.289(2, 70) | 0.282 | 0.036 | _ |
|  | Magnitude × Arrangement × Hemisphere | 0.938(8, 280) | 0.485 | 0.026 | _ |
| Latency | Arrangement | 0.633(1.462, 51.179) | 0.488 | 0.018 | 0.755 |
|  | Magnitude | 0.441(4, 140) | 0.779 | 0.012 | _ |
|  | Hemisphere | 0.062(1, 35) | 0.805 | 0.002 | _ |
|  | Magnitude × Arrangement | 0.8444(5.340, 186.909) | 0.564 | 0.024 | 0.802 |
|  | Magnitude × Hemisphere | 0.074(4, 140) | 0.990 | 0.002 | _ |
|  | Arrangement × Hemisphere | 0.106(2, 70) | 0.899 | 0.003 | _ |
|  | Magnitude × Arrangement × Hemisphere | 0.822(5.360, 187.595) | 0.584 | 0.023 | 0.805 |

**Table S4.** Repeated measure ANOVA results for peak amplitude and latency of N1 component over parieto-temporal electrodes (including: T7, TP7 and P7 on the left side and T8, TP8 and P8 on the right side).

|  | Factors | *F* (df, error) | *P* | *ɳ_p_^2^* | ε |
| --- | --- | --- | --- | --- | --- |
| Amplitude | Arrangement | 0.443(2, 70) | 0.644 | 0.012 | _ |
|  | Magnitude | 0.686(4, 140) | 0.603 | 0.019 | _ |
|  | Hemisphere | 0.827(1, 35) | 0.369 | 0.023 | _ |
|  | Magnitude × Arrangement | 0.437(8, 280) | 0.898 | 0.012 | _ |
|  | Magnitude × Hemisphere | 0.279(3.007, 105.262) | 0.841 | 0.008 | 0.830 |
|  | Arrangement × Hemisphere | 0.650(2, 70) | 0.525 | 0.018 | _ |
|  | Magnitude × Arrangement × Hemisphere | 0.599(8, 280) | 0.778 | 0.017 | _ |
| Latency | Arrangement | 0.822(2, 70) | 0.444 | 0.023 | _ |
|  | Magnitude | 0.551(4, 140) | 0.699 | 0.015 | _ |
|  | Hemisphere | 0.29(1, 35) | 0.865 | 0.001 | _ |
|  | Magnitude × Arrangement | 1.792(8, 280) | 0.078 | 0.049 | _ |
|  | Magnitude × Hemisphere | 1.981(4, 140) | 0.101 | 0.054 | _ |
|  | Arrangement × Hemisphere | 0.638(2, 70) | 0.531 | 0.018 | _ |
|  | Magnitude × Arrangement × Hemisphere | 1.067(5.571, 194.998) | 0.382 | 0.030 | 0.843 |


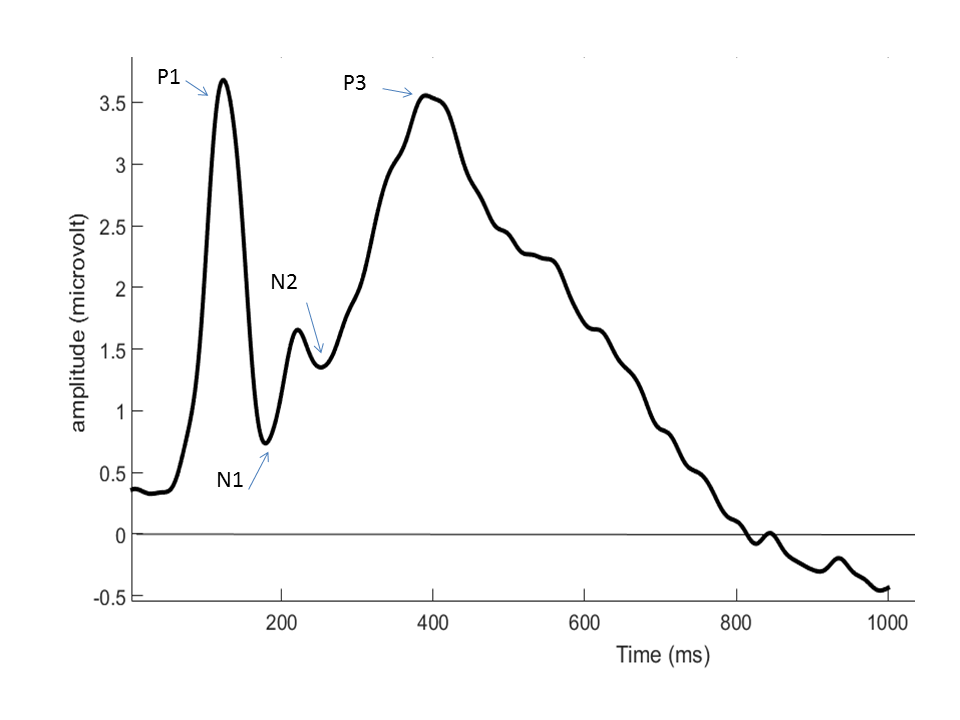


*Figure S1.* Grand average ERP waveform over right posterior ROI including P2, P4, PO4 and PO6 electrodes

***2.* Source modeling using minimum norm imaging**

Minimum norm imaging estimates the sources as the solution to a linear imaging problem. This method finds a cortical current source density (CSD) image that approximately fits the data when mapped through the forward model. These z-score baseline normalized maps were calculated for each arrangement and magnitude separately. Since subsequent numbers were included in the experiment, for magnitude, only the smallest and the largest numerosities were included (8 and 12 dot sets). Then a time-point-by-time-point *t*-test was performed to see whether there was a significant (*p*<.05) difference between each of the two conditions of arrangement/magnitude in our time intervals of interest. Here the FDR correction was again controlled across all time-points and signal comparisons using the Brainstorm software. These time intervals were selected according to significant time intervals in ERP data. Since a point-by-point *t*-test was performed to compare the sources in different conditions, the results show the difference of activity between areas. This is also pointed out in related figures. According to these results, the source difference between arrangements were not significant before 400 ms post stimulus (*p*>.05). These non-significant differences are presented in Figure 2 and Figure 3 for 230-300 and 320-370 ms post stimulus. However, after 400 ms the difference was highly significant (*p*<.01). In the case of magnitude, the significant difference was poorly observed over the right frontal region at 500 to 600 ms after stimulus (*p*<.07). The difference between each of the two conditions for each factor was calculated afterward by subtraction and then the sources were averaged across each time interval of interest. Figure 2 illustrates the difference between current source density calculations for irregular and quadrangular arrangements from 230 to 300 ms post stimulus. This process was repeated for other significant time intervals (see Figure 3 and Figure 4). For late processing stages (after 400 ms) the sources were averaged across consecutive 100 ms time windows as we did in the ERP analysis. Results showed a constant location of differences among arrangements in the entire time window from 400 to 1000 ms (Figure 4). This is in line with our statistical results which showed no interaction between arrangement and time and no significant change in ERP activity was shown during this time interval (see Figure 6 in the manuscript). Considering the magnitude, the effect of magnitude on sources at the late stage of processing was investigated by calculating the source difference over 500 to 600 ms between numbers 8 and 12 (see Figure 5). This time window was selected according to the more obvious significant effect of magnitude over time in the ERP analysis (see Figure 8b in the manuscript).

Irregular - Quadrangular Irregular- Hexagonal


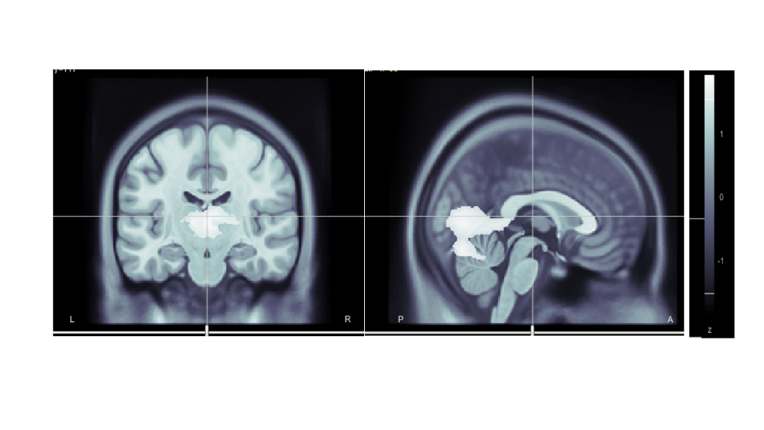

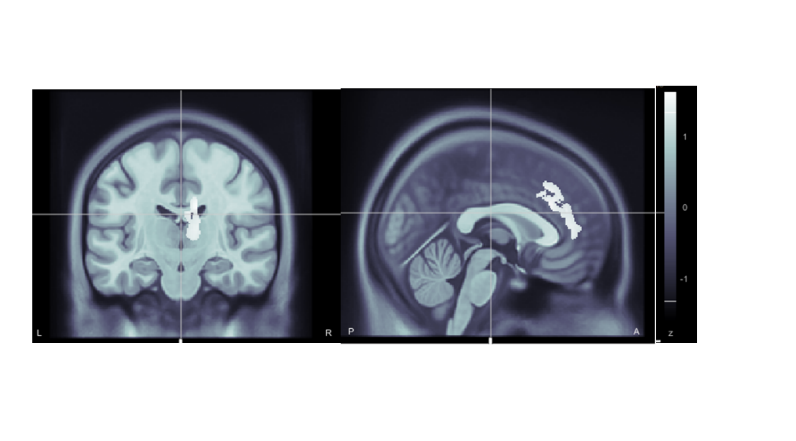


Hexagonal – Quadrangular

**Z**

**1**


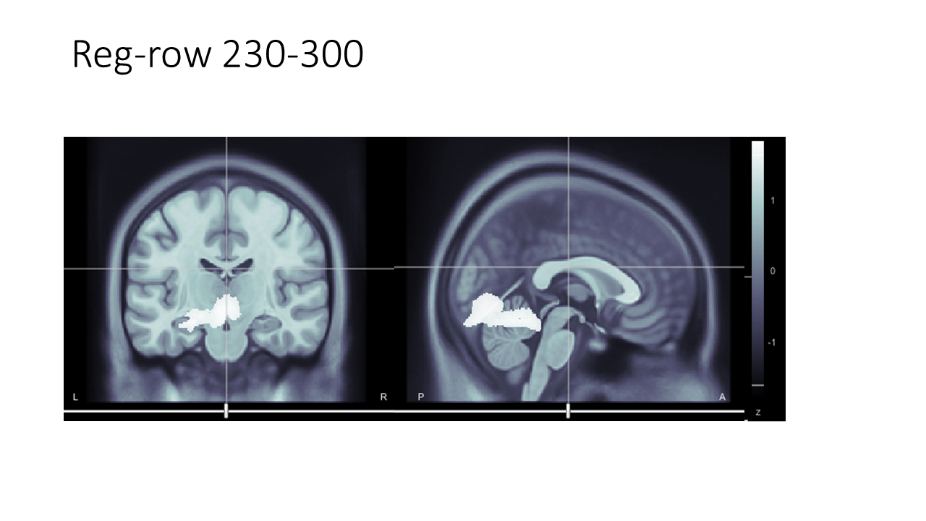

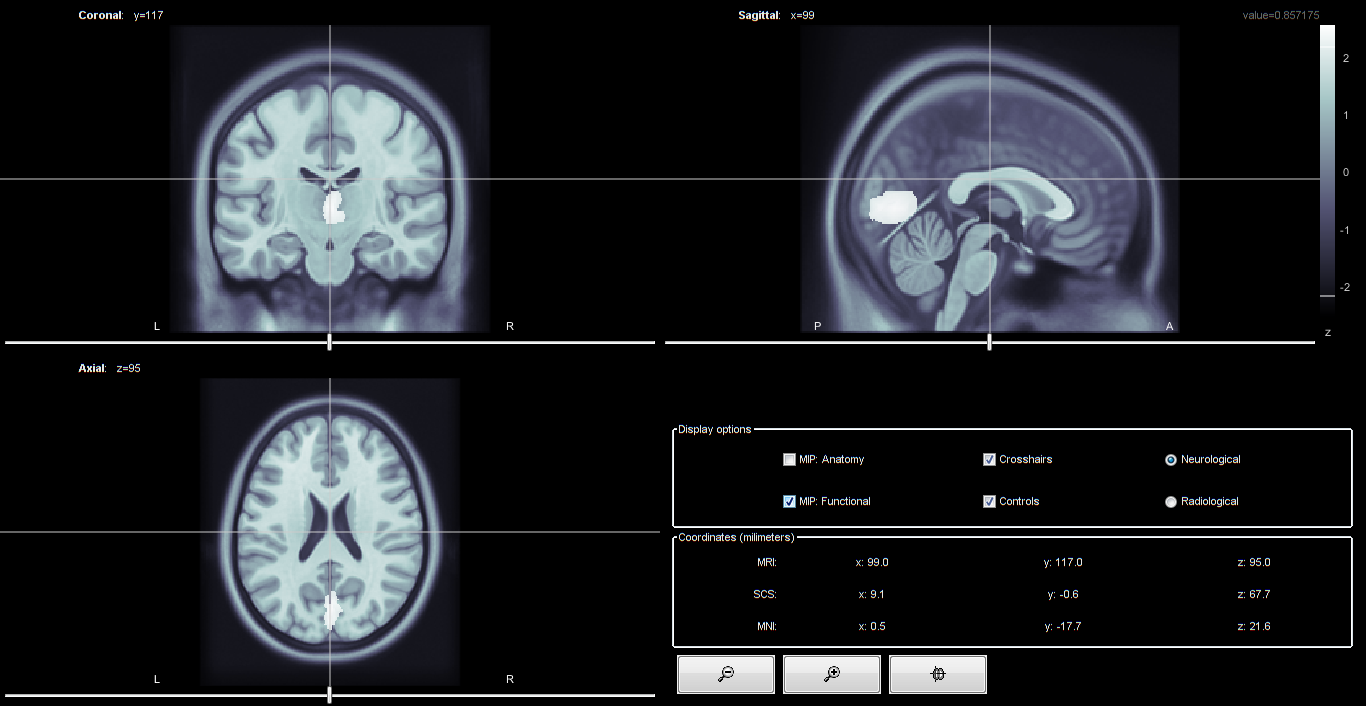


**P**

**A**

**R**

**Lorrespondence**

**0**

**-1**

*Figure S2.* The difference in current source density maps among three arrangement conditions. Activation was averaged across all participants and integrated over 230 to 300 ms post stimulus. P value analysis did not show significant differences between conditions at this time interval.

Irregular – Quadrangular Irregular- Hexagonal


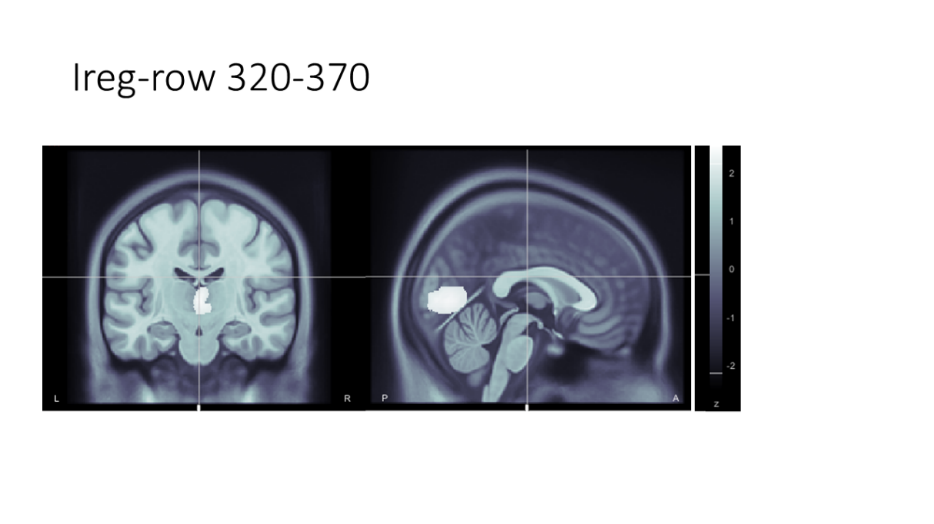

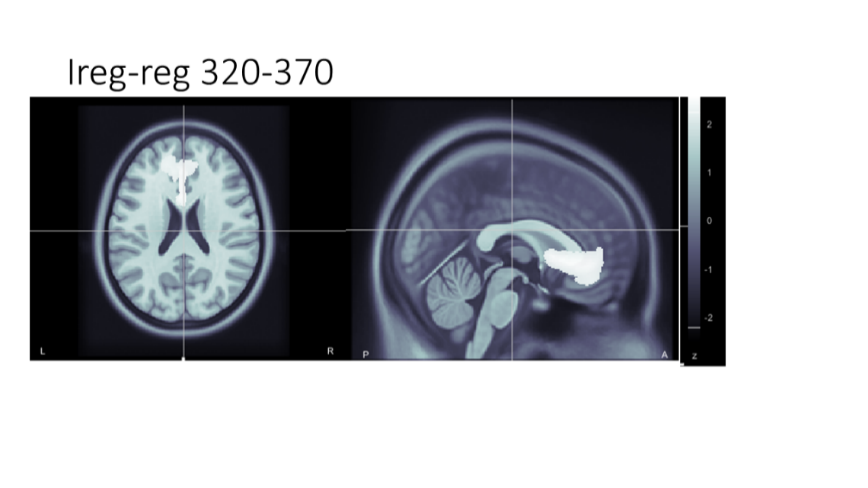


Hexagonal – Quadrangular

**2**

**Z**


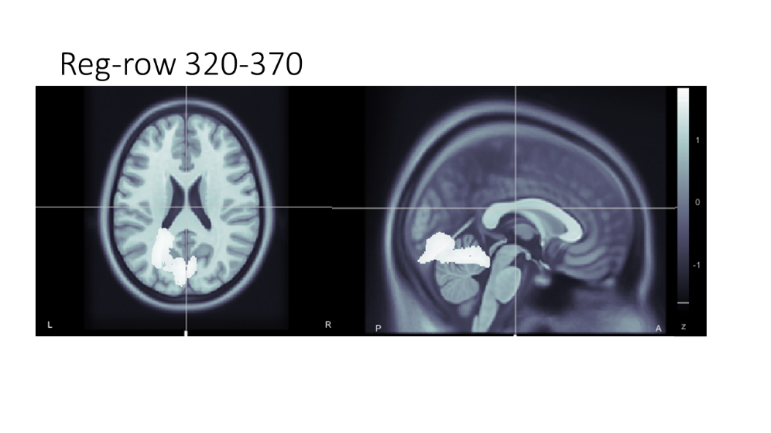

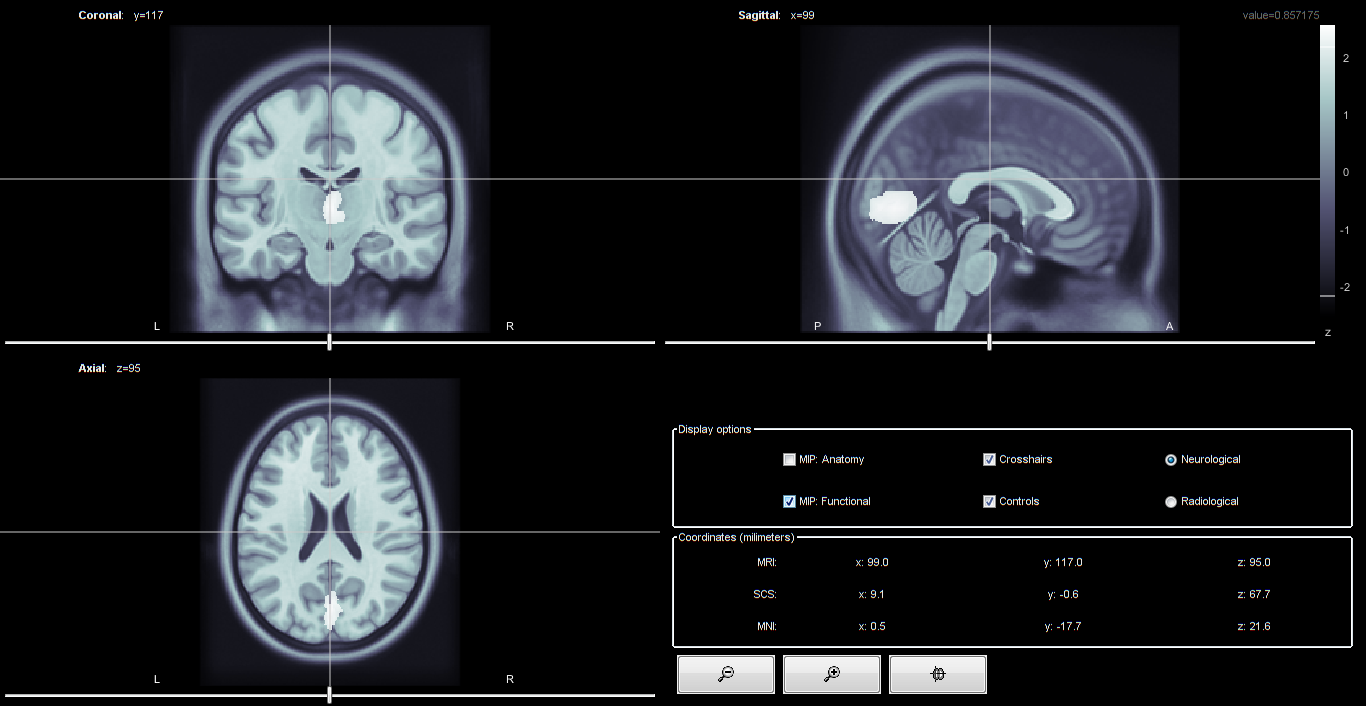


**P**

**A**

**R**

**Lorrespondence**

**0**

**-2**

*Figure S3.* The difference in current source density maps among three arrangement conditions. Activation was averaged across all participants and integrated over 320 to 370 ms post stimulus. P value analysis did not show significant differences between conditions at this time interval.

Irregular – Quadrangular Irregular- Hexagonal


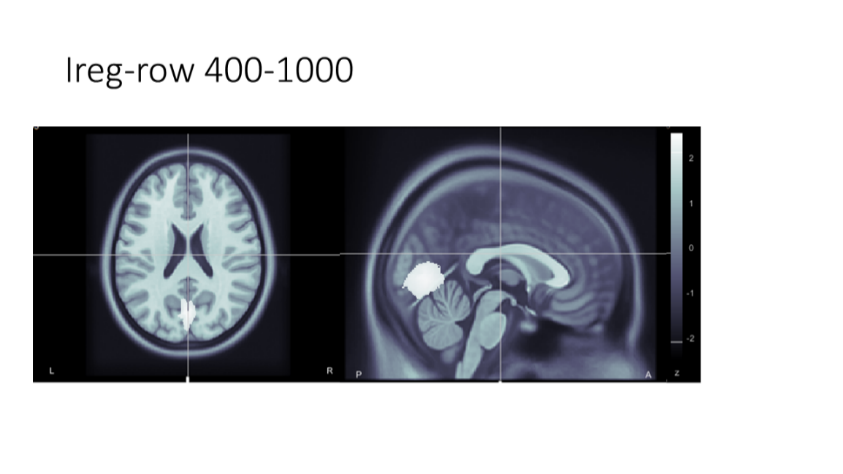

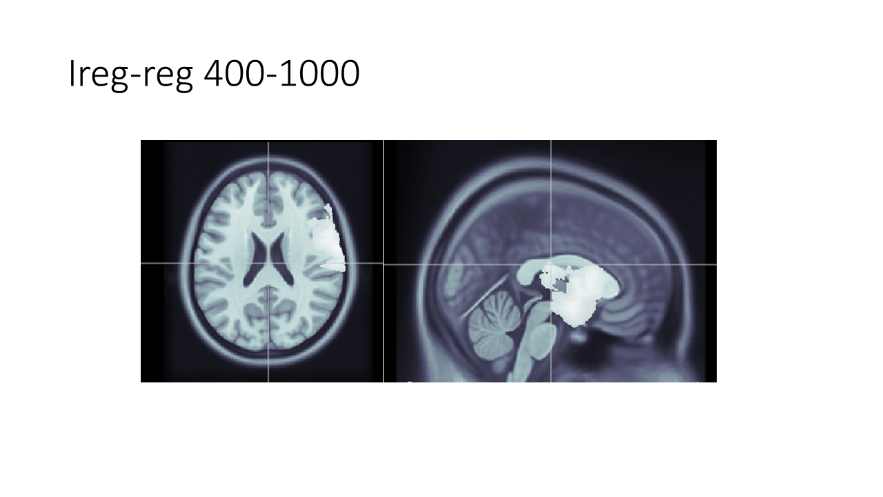


Hexagonal – Quadrangular

**2**

**Z**


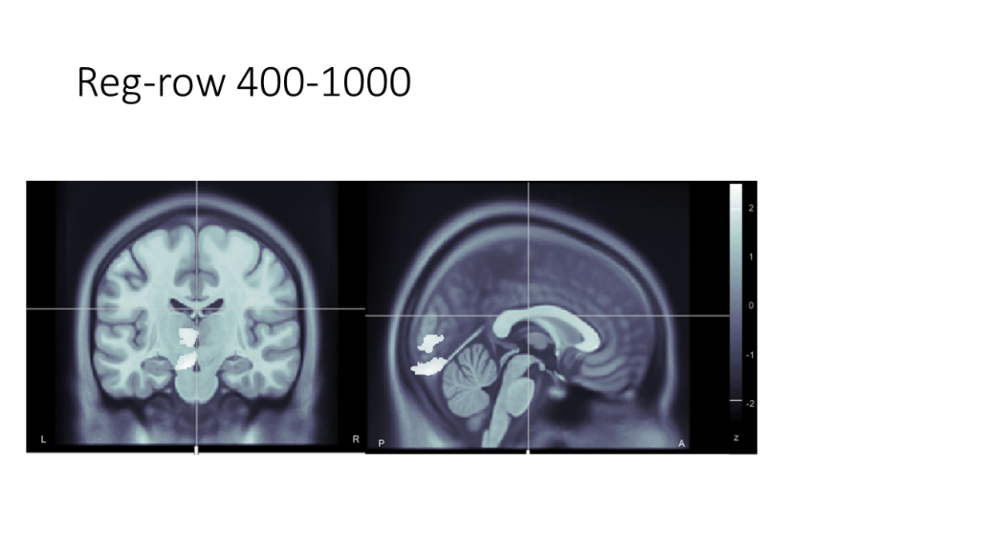

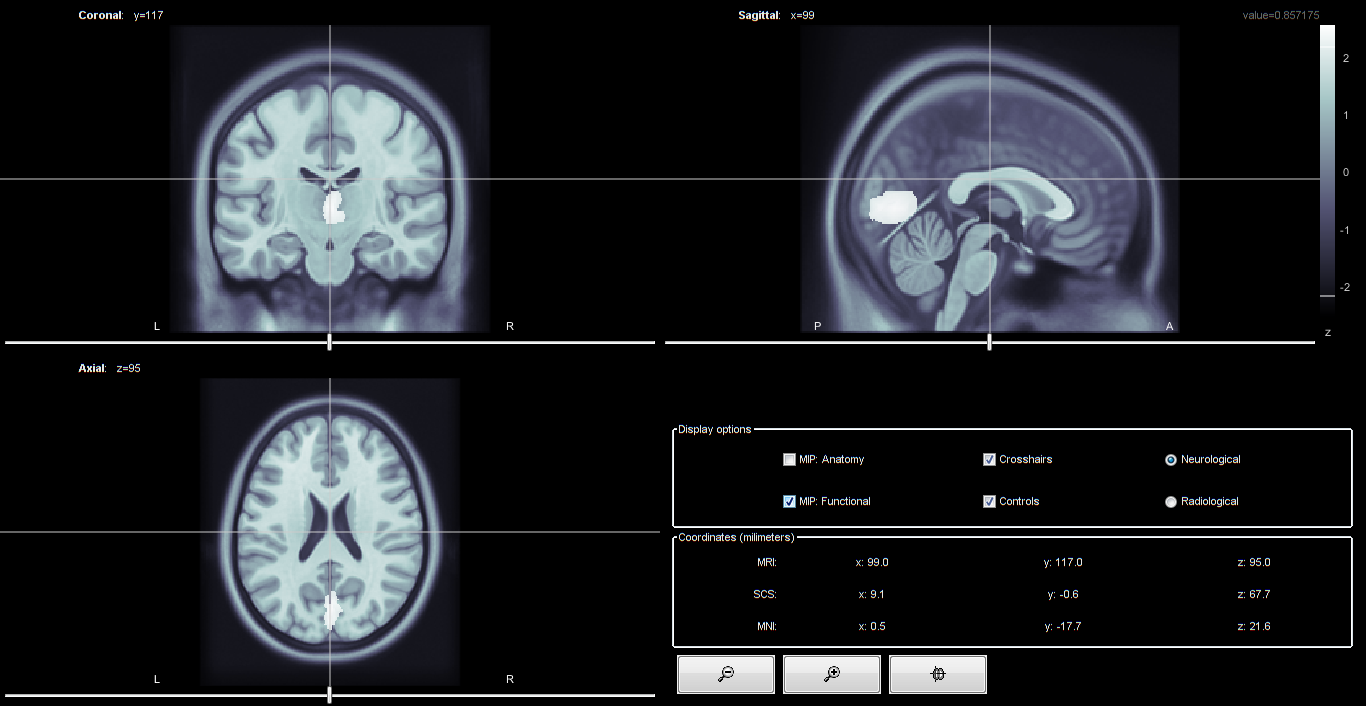


**Aorrespondence**

**Porrespondence**

**Rorrespondence**

**Lorrespondence**

**-2**

**0**

*Figure S4.* The difference in current source density maps among three arrangement conditions during higher level processing of enumeration. Activation was averaged across all participants and integrated over consecutive 100 ms time intervals from 400 to 1000 ms post stimulus. Due to similar results, only one time interval (400-500 ms) is graphed.

12-8

**Z**

**2**


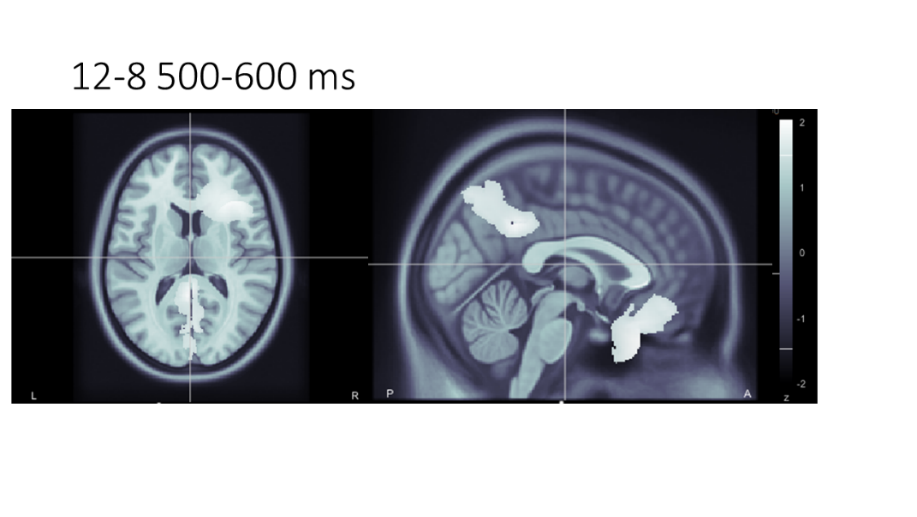

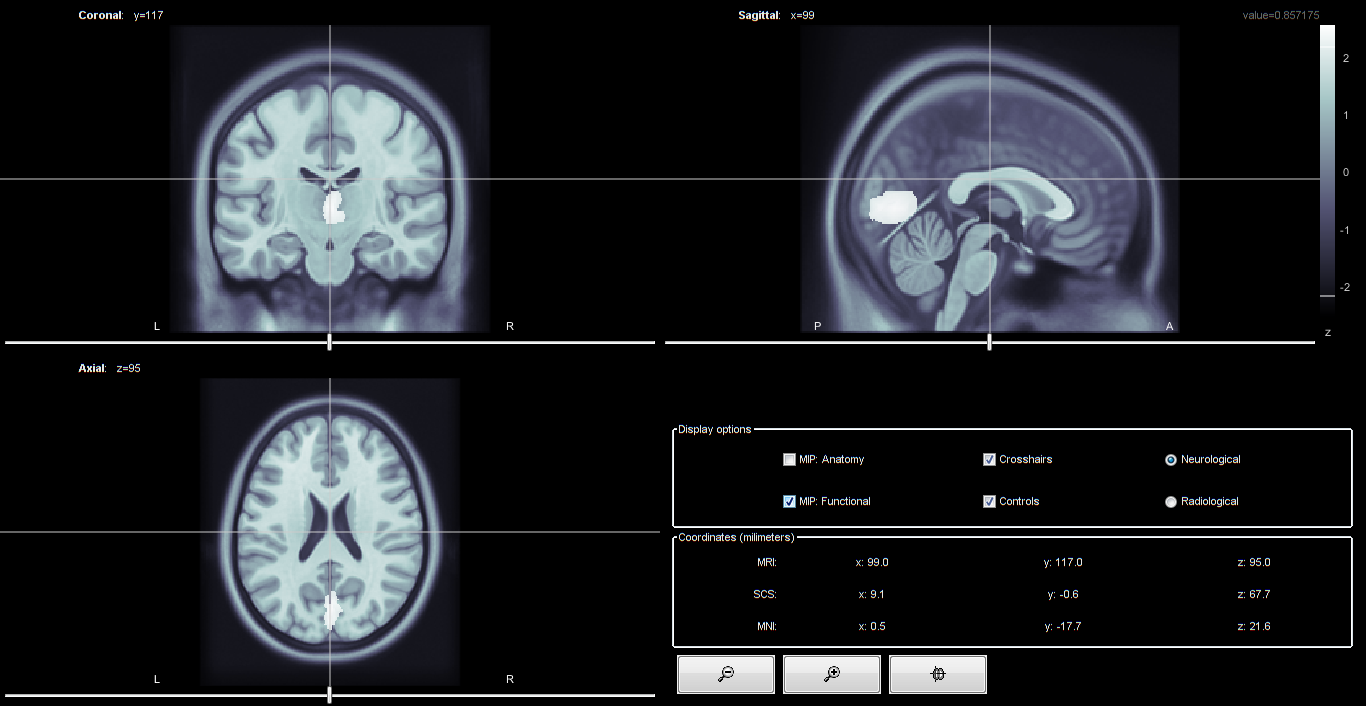


**Aorrespondence**

**Rorrespondence**

**Porrespondence**

**Lorrespondence**

**0**

**-2**

*Figure S5.* The difference in current source density maps between the largest and smallest magnitude conditions, 8 and 12, during higher level processing of enumeration. Activation was averaged across all participants and integrated over 500-600 ms post stimulus.

|  | Irregular | Hexagonal | Quadrangular |
| --- | --- | --- | --- |
| 8 | 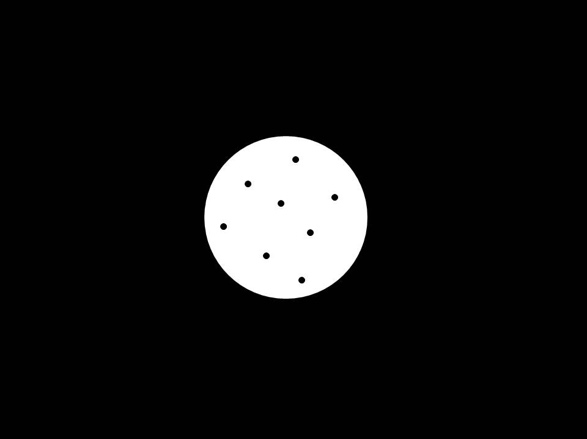 | 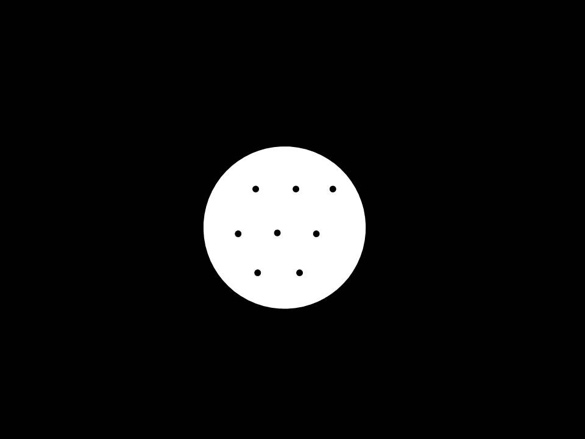 | 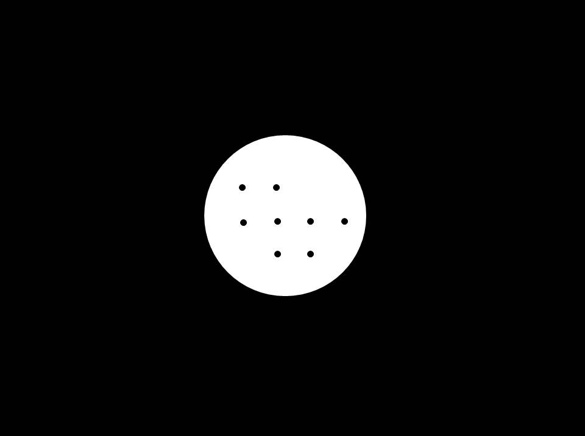 |
| 9 | 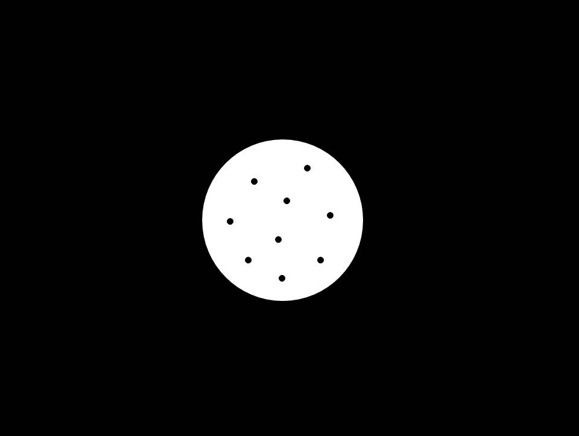 | 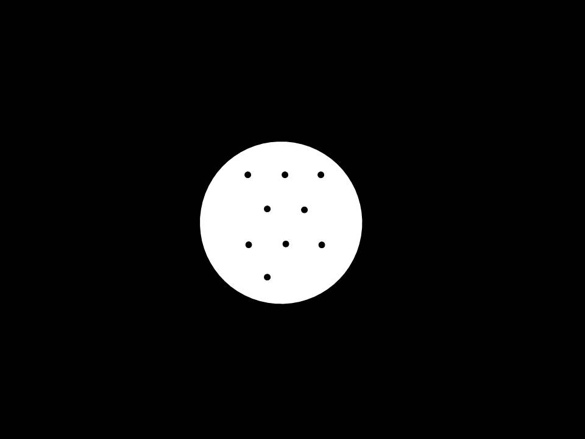 | 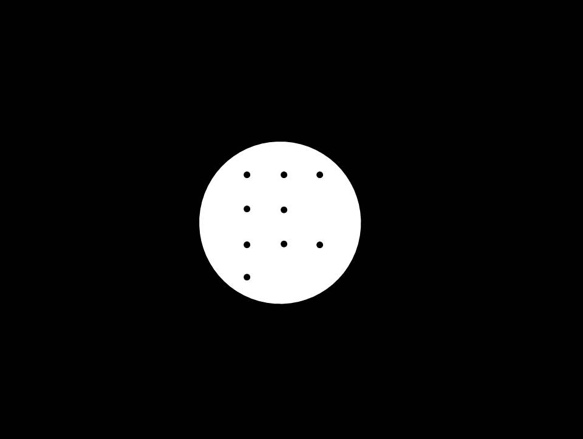 |
| 10 | 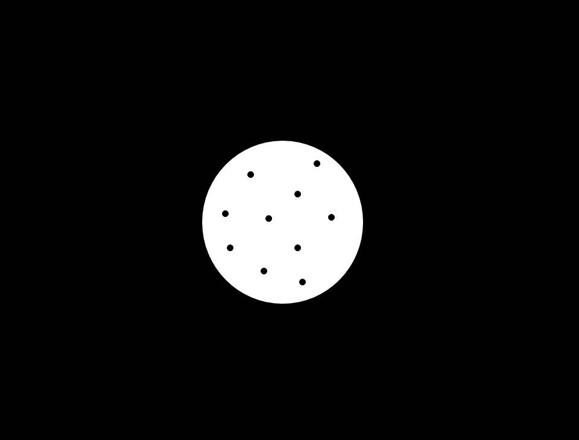 | 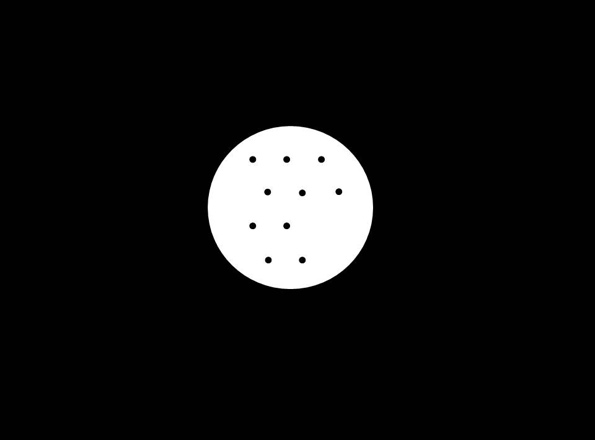 | 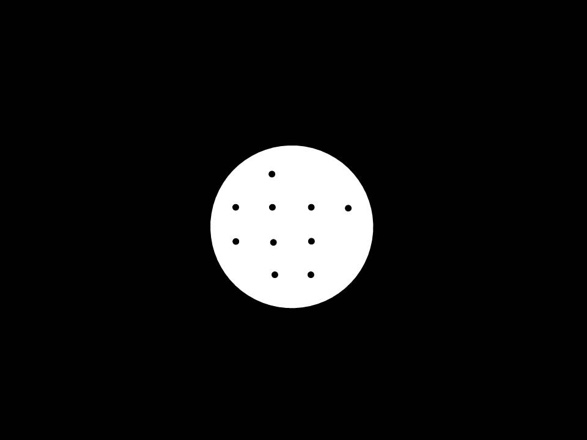 |
| 11 | 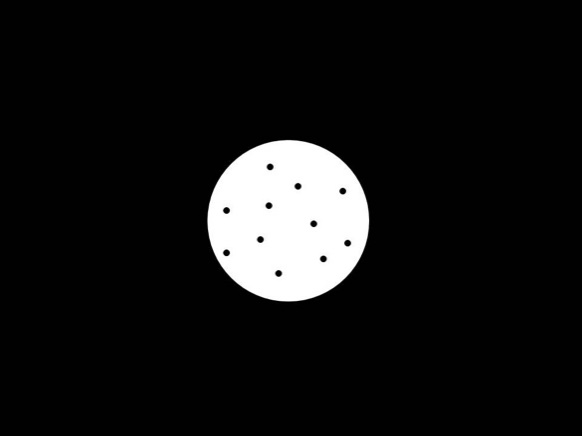 | 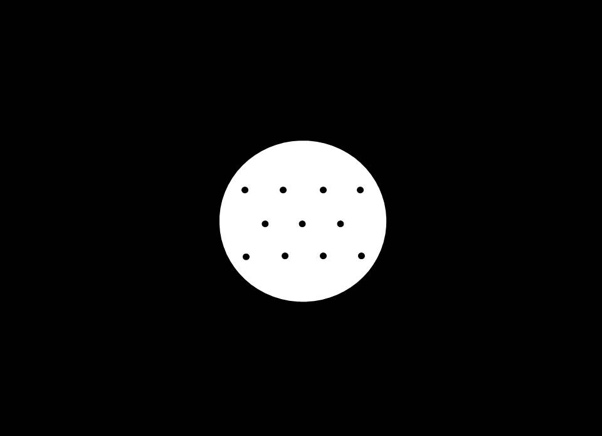 | 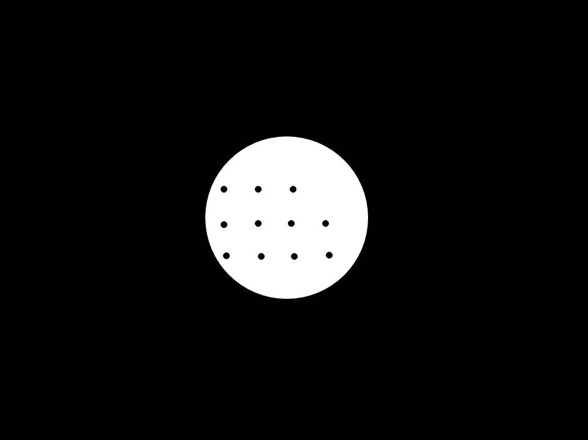 |
| 12 | 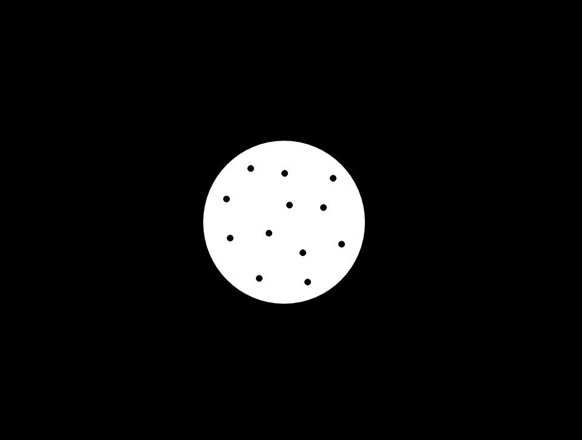 | 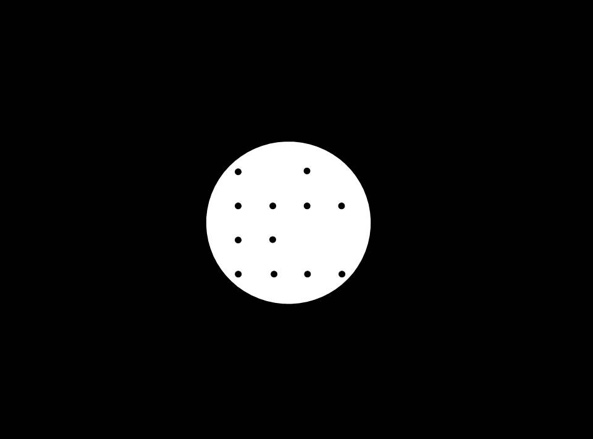 | 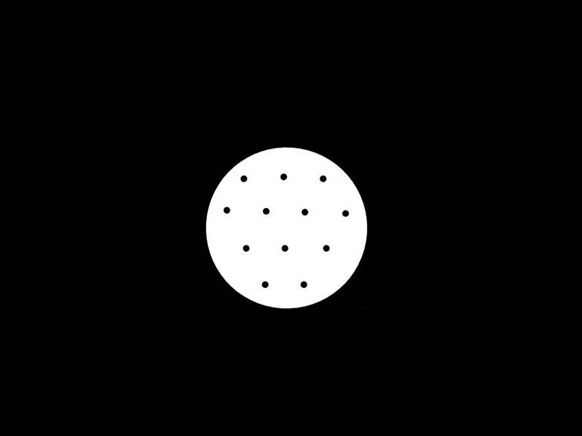 |

*Figure S6*. An overview of typical 3x5 stimulus arrays. From each “number x arrangement” category, one slide sample is presented.
